# Design, Synthesis, and Antifungal Activity of 4-Amino Coumarin Based Derivatives

**DOI:** 10.3390/molecules27092738

**Published:** 2022-04-24

**Authors:** Lu Xu, Jinmeng Yu, Lu Jin, Le Pan

**Affiliations:** College of Chemistry and Chemical Engineering, Xinjiang Agricultural University, Urumqi 830052, China; xl281644038@163.com (L.X.); yujinmeng321@163.com (J.Y.)

**Keywords:** succinate dehydrogenase, inhibitor, 4-amino coumarin, fungicide, antifungal activity, molecular docking

## Abstract

A series of 30 succinate dehydrogenase inhibitors (SDHIs) of 4-amino coumarin-based derivatives were designed and synthesized. According to the analysis of fungicidal activity in vitro, most of the compounds expressed broad-spectrum antifungal activity against four plant pathogenic fungi (*Alternaria alternata, Alternaria solani, Fusarium oxysporum,* and *Botrytis cinerea*) using the mycelium growth inhibition method. The results showed that compounds **3n** with the group of 2-ene-3-methyl-butyl and **4e** with the group of 2-bromo-1-oxo-hexyl displayed excellent activity against *Alternaria alternata* and *Alternaria solani*, with EC_50_ values of 92~145 μg/mL. Molecular docking showed that the inhibitor **3n** was completely locked into the cavity of SDH, forming a conventional hydrogen bond interacting with the amino acid residue TYR58. The present work indicates that these derivatives would serve as novel potential fungicides targeting SDH.

## 1. Introduction

Plant disease caused by phytopathogenic fungi is a worldwide threat to food security and crop protection. Antifugal agrochemicals are crucial to fight against plant pathogens. Succinate dehydrogenase (SDH), the only enzyme involved in both the respiratory chain and the tricarboxylic acid (TCA) cycle, plays an important role in the mitochondrial electron transfer chain. Fungicides that can inhibit SDH have been classified and named as SDH inhibitors (SDHIs) by the Fungicide Resistance Action Committee [1]. The unique mode of SDHIs is to block the TCA cycle, inhibiting the respiratory chain of fungi and leading to fungi death. Since carboxin was first commercially applied in 1969, more than 20 SDHIs fungicides have been commercialized, most of which have an amide bond [2]. The research of SDH inhibition of some carboxamide fungicides has indicated that the carboxamide competes with ubiquione rather than succinate. Here, the carbonyl oxygen of the carboxamide can form hydrogen bonds with two key amino acid residues (TRP and TYP) [3].

Despite advances in the development of SDHIs for fungicides, there has been rapid development in pathogen resistance due to the unrestricted usage of fungicides. All commercial fungicides of SDHIs, possessing a similar scaffold, can easily generate cross-resistance [4]. In addition, some reported fungicides of SDHIs have also retained their essentially common amide bond, and other aspects of structural diversity were tested [5,6,7]. For example, Wu and coworkers studied N-(pyridine-4-yl)-1-phenyl-5-trifluoromethyl-1H-pyrazole-4-carboxamide derivatives as potential SDHIs [8,9]. The results showed that some compounds exhibited excellent antifungal activity against four phytopathogenic fungi (*G. zeae*, *F. oxysporum*, *C. mandshurica*, and *P. infestans*). Zhao et al. reported on the antifungal activity of novel longifolene derived diacylhydrazine compounds [10]. A combination of experimental and theoretical investigations revealed that the relative inhibitory rate is over 70% for some compounds. Systemically using some pyrazole-containing carboxamides as potent SDH inhibitors was reported recently [11]. Therefore, it is urgently required to develop some novel SDHIs with different scaffolds in order to prevent cross-resistance.

It would be very interesting and challenging to design and find new compounds possessing antifungal properties for application in agriculture or forestry. The heterocyclic structure is a key feature in natural products and synthetic compounds used as medicines or pesticides [12,13,14]. Of these, coumarin is related to a large class of natural compounds [15,16]. Molecules with a coumarin moiety have displayed great potency in the treatment of diseases [17,18,19], and over 1300 coumarins have been identified principally as secondary metabolites in plants, fungi, and bacteria [20,21,22,23]. In addition, synthetic coumarins with a wide variety of groups at the C-3, C-4, and C-7 positions have been screened for their biological properties.

**Scheme 1:**
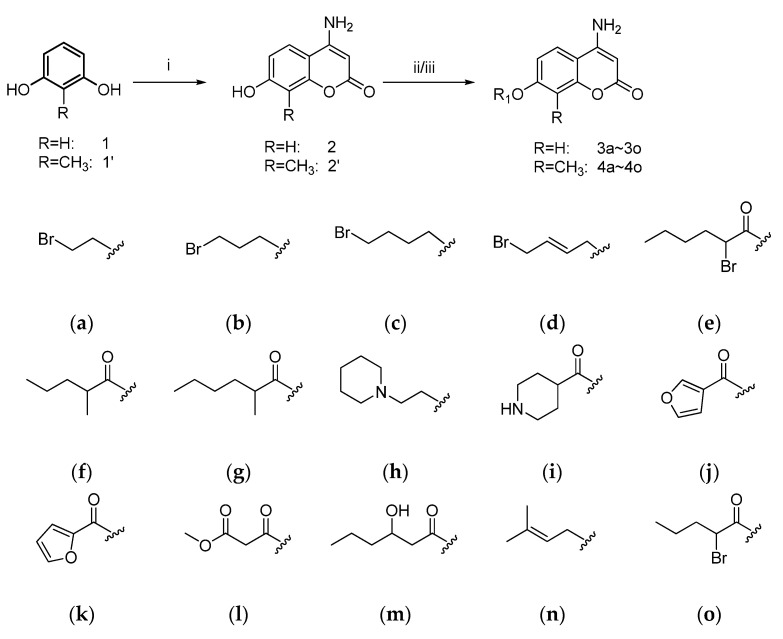
Synthesis routes of compounds **3a**–**3o** and **4a**–**4o.** R_1_ refers to the substituent labeled (**a**–**o**). (i) Cyanoacetic acid, anhydrous ZnCl_2_ and HCl gas, at room temperature, 5 h; (ⅱ) substitutes, anhydrous K_2_CO_3_, TBAB, acetone, reflux; (ⅲ) substitutes, thionyl chloride, acetone, reflux.

Inspired by our previous work, in which some C7-substituted umbelliferone derivatives (UDs) exhibited excellent fungicidal activity [24,25], we expanded the scope and designed the title compounds to screen for antifungal activity. The amino group, an electron-donating substituent, can affect the molecular orbitals, electron transfer, and molecular interaction. To our knowledge, the biological activity of coumarine substituted by an amino group has been rarely reported, and especially 4-amino derivatives. The preliminary molecular docking experiments indicated that the core structure could match with the ubiquinone (UQ)-site pocket and enter into it. As shown in Scheme 1, based on the core structure of 4-amino coumarines, we designed flexible groups to be installed at the 7-hydroxy position for fitting with the target site. In total, 30 derivatives were synthesized, and we explored their antifungal activity on *Alternaria alternata*, *Alternaria solani*, *Fusarium oxysporum*, and *Botrytis cinerea*. The in vitro inhibitory activity was evaluated to screen a novel SDHI. The typical inhibition with mycelium growth in solid medium was used to study the primary antifungal activities of the four pathogens in this work.

## 2. Results and Discussion

### 2.1. Synthesis

The synthesis of 4-amino coumarine derivatives is outlined in Scheme 1. In total, 30 derivatives were obtained by procedures i~iii in moderate to high yields. The structures have been confirmed by MS, ^1^H NMR, and ^13^C NMR. Details of the procedures and characterization are provided in the experimental section. As far as we know, 28 compounds, except **3a** [18] and **4k** [26,27], are reported for the first time herein. The key precursor 7-hydroxy-4-amino coumarin (denoted as **2**) was formed by the reported procedure i [18]. The ^1^H NMR peaks of 7.73, 6.78 and 6.67 Hz were assigned to the signals of H at the C-5, C-6, and C-8 sites in **2**, which were inconsistent with the reported data [18]. Note that the signals of hydroxy, amino, and H at C-3 would be missed due to the isotopic exchange of the solvent (CD_3_OD). After installing the substituent at the C-7 position, 4-amino-7-(2-bromo ethoxy)-2H-chromen-2-one (**3a**) indicated the signals of H at C-5, C-6, C-8 and C-3, as well as two positions of the side chain, which were 7.83, 6.96, 6.89, 5.28, 4.40, and 3.7 Hz, respectively, according to ^1^H NMR, and these were also inconsistent with the reported data [18]. The corresponding [M + H]^+^ peak of 284.98 was found in the mass spectrum. Our observed melting point was 190 °C, which is lower than the reported value (222 °C) [18]. The mass (285 of M^+^) of **4k** was reported without any other data on the characterization [26,27]. For other products, we have carefully checked and confirmed the signals.

Although 30 derivatives are not enough, we have considered the degree of rotation (length of chain, branched chain, and ring) and the bioactive groups, such as halogen, carbonyl, and amine group, etc. Moreover, a methyl at the C-8 position was taken into account to compare the bioactivity in parallel.

### 2.2. Screening of Antifungal Activity

As we were limited by our experimental conditions, only four typical pathogens (*Alternaria alternata, Alternaria salani, Fusarium oxysporum,* and *Botrytis cinerea*) were prepared and used to test the antifungal activity with the obtained 30 compounds at 200 µg/mL. To evaluate the tolerance of the antifungal activity, the duration times of 48, 72, and 96 h were considered, respectively. It was found that the inhibition rate had slightly changed with a longer time. Here, we only discuss the inhibition rate over 96 h, and other results are shown in the Supplementary Materials.

Note that the substituent groups at 7-hydroxy are crucial in compounds without the 8-methyl group (**3a**–**3o**), because **2** exhibited very poor activity against the four tested pathogens. As shown in Table 1, after the 7-hydroxy was modified by substituent groups, the antifungal activities of most of the derivatives were successfully improved. From the comparative results, it can be seen that most of the synthesized compounds exhibited more antifungal activity against *A. Salani* and *A. Alternata* than *B. Cinerea* and *F. Oxysporum*. For example, compounds **3c** and **4c**, with a flexible bromo-n-butyl chain, showed moderate bioactivity (20~30%) for *A. Salani* and *A. Alternata*, whereas they had little effect on *B. Cinerea* and *F. Oxysporum.* Therefore, we mainly discuss the antifungal activity against *A. Salani* and *A. Alternata* hereafter. Interestingly, the results indicate that both the length of the side chain and the 8-methyl group would account for the inhibitory activity. As shown in Table 1, the antifungal activity of **3a** (possessing a 2-carbon chain) was higher than tose of **3b** (possessing a 3-carbon chain) and **3c** (possessing a 4-carbon chain), revealing that a compound bearing a short side chain would show high inhibitory activity. On the contrary, as for the 8-methyl group-installed compounds (Table 2), **4c** (possessing a 4-carbon chain) was more active than **4a** (possessing a 2-carbon chain) and **4b** (possessing a 3-carbon chain), indicating that the long length of the chain would result in high activity. Importantly, this simple rule as stated above was also relevant to other compounds, such as **3e** (possessing a six-carbon chain) vs. **3o** (possessing a five-carbon chain), and **4e** (possessing a six-carbon chain) vs. **4o** (possessing a five-carbon chain). Namely, **4e,** including the 8-methyl group, has higher antifungal activity than **4o**, whereas the activity of **3e** was lower than that of **3o** without the 8-methyl group. Both **3o** and **4e** expressed somewhat strong antifungal activity against *B. Cinerea* and *F. Oxysporum*. In order to assess the lipophilicity, the corresponding log*P*_O/W_ was calculated for all tested compounds (see details in the Supplementary Materials). The calculated values of log*P*_O/W_ showed that the 8-methyl group could improve the lipophilicity, and that the lipophilicity would gradually increase with the growth of the side chain. In this sense, it has been difficult to provide a unified relationship between the different activities originating from the length of the side chain and the trend of the lipophilicity. We have deemed that the inhibitory activity might be influenced by the interaction of the docking target. Furthermore, if the cyclic fragment was included in the substituent, our results indicate that the antifungal activity would be poor, such as with **3h**, **4h**, **3i**, **4i**, **3j**, **4j**, **3k**, and **4k**.

**Figure 1:**
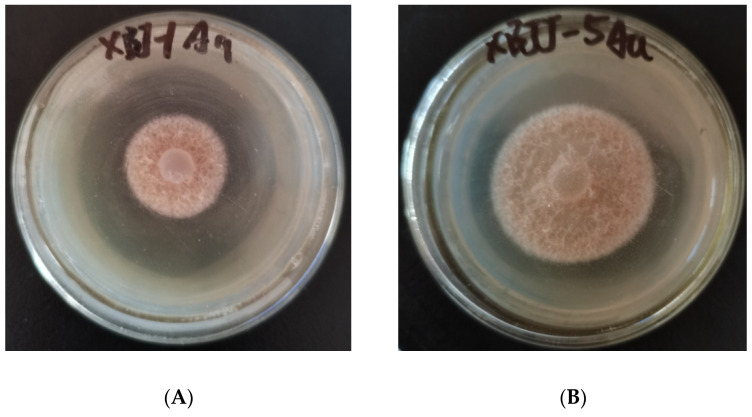

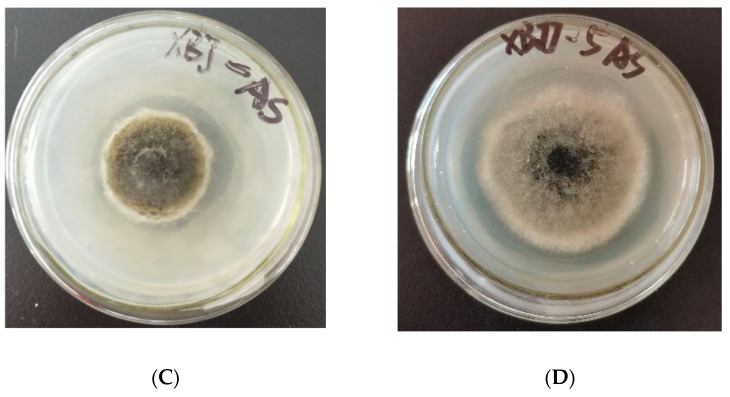
In vitro inhibition of mycelial growth of *A. Alternata* and *A. Salani* by compounds **3n** and **4n**. (**A**) **3n** at 200 μg/mL for *A. Alternata*; (**B**) **4n** at 200 μg/mL for *A. Alternata*; (**C**) **3n** at 200 μg/mL for *A. Salani*; (**D**) **4n** at 200 μg/mL for *A. Salani*.

It was found that compound **3n** demonstrated the highest antifungal activity out of the 30 molecules tested against the four fungi. Interestingly, the substituent group of 3-methyl-2-butenyl in compound **3n** is a common moiety in coumarin-based natural products, such as osthol, pyranocoumarin after intramolecular condensation, and peucedanin. Similarly, compared with *B. Cinerea* and *F. Oxysporum* (ca. 40%), *A. Salani* and *A. Alternata* (ca. 80%) suffered about two-fold inhibition by compound **3n**. After the methyl group was installed at the C-8 position (compound **4n**), the inhibitory activity dramatically reduced by half, showing a negative effect. The pictures of inhibition given by the mycelium growth rate method for the representative compounds **3n** and **4n** are shown in Figure 1. From the size of growth, we can see that **3n** obviously showed stronger inhibition than **4n** against both *A. Salani* and *A. Alternata.* On the contrary, some other compounds showed a positive effect after the methyl group was installed at the C-8 position. For example, the average inhibitory activity of compound **3e** containing 2-bromo-1-oxo-hexyl chain was about 11%, except for the 21% achieved by *A. Alternata*, whereas the 8-methyl group in compound **4e** yielded an averaged inhibitory activity, which increased by about four-fold to 40%. In this sense, as they vary with the substituent groups at the 7-hydroxy position, the effects of the 8-methyl group would not be clear at a glance.

As mentioned above, of the 30 tested compounds, **3n** and **4n** showed good activity, with inhibitory rates of above 70% and 40% against *A. Salani* and *A. Alternata*, respectively. In addition, compounds **4e** and **4g**, containing 1-oxo-2-substituent-hexyl, showed moderate antifungal activity (>40%) against *A. Salani* and *A. Alternata.* Accordingly, compounds **3e** and **3g** without the 8-methyl group should be compared in further EC_50_ studies. Note that the bromide group would be more active than methyl in the moiety of 1-oxo-2-substituent-hexyl, namely, **3e** > **3g** and **4e** > **4g**.

### 2.3. Antifungal Activity Affected by Concentration (EC_50_ Value)

Furthermore, in order to comprehensively study the antifungal activity, a series of concentrations of the effective compounds were examined for the evaluation of their EC_50_ values. As discussed above, three compounds (**3n**, **4e** and **4g**) showed fair to good activity, and were taken as candidates to test their EC_50_ values at concentrations of 200, 100, 50, 25, and 12.5 μg/mL over 96 h, in order to determine their antifungal activity against *A. Salani* and *A. Alternata*. In order to compare the antifungal effect with and without methyl groups at the C8 position, three relevant compounds, **4n**, **3e** and **3g**, were also chosen to evaluate EC_50_ values. Three replicates were applied in each treatment, and EC_50_ (μg/mL) values were obtained by probit analysis using the SPSS program. As shown in Table 3, compounds **3n** and **4e** demonstrated the best antifungal activities against *A. Salani* and *A. Alternata.* The corresponding EC_50_ values of **3n** were 107.4 and 96.7 μg/mL, while the EC_50_ values of **4e** were 144.5 and 92.1 μg/mL. It was worth noting that the competitive candidate **4n** lagged in the EC_50_ examination. Finally, we screened out two potent SDH inhibitors, **3n** and **4e**, in this work.

### 2.4. Molecular Docking

**Figure 2:**
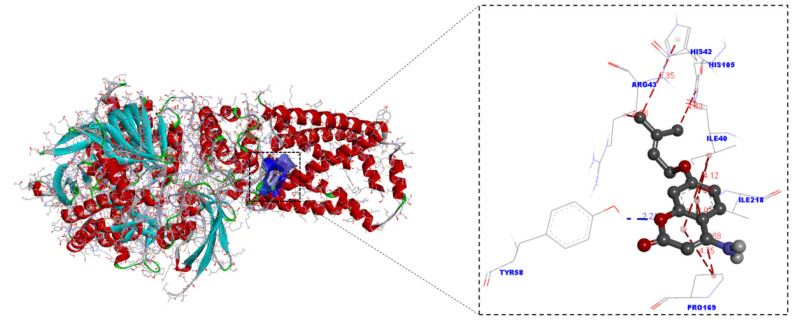
Binding mode and the interaction of inhibitor **3n** docking with SDH (PDB code: 1YQ3).

As stated above, compound **3n** has high activity, and we therefore selected this compound as the docking ligand. To study the binding details of compound **3n** with succinate dehydrogenase, a docking study was performed using the Autodock Vina code [28]. Based on the X-ray crystal structure (PDB code: 1YQ3) [29], the UQ-site was considered as the docking cavity. As shown in Figure 2, the coumarin moiety was completely incorporated into the substrate cavity, forming a conventional hydrogen bond interacting with the amino acid residues TYR58. The corresponding hydrogen bond distance was 2.21 Å. Besides this, compound **3n** was surrounded by the hydrophobic residues HIS42, ARG43, HIS105, ILE40, ILE218, and PRO169. Note that the substituent was very crucial for docking. The substituent not only controlled the binding mode, but also had an effect on the interaction energy. For **3n**, due to the suitable size of the substituent and the strong interaction with residues, the moiety of the substituent was considered as the head in the docking with SDH, differently from some other tested structures, in which the substituent groups acted as the tail.

## 3. Conclusions

A series of novel coumarin derivatives containing an amino group were designed, synthesized, and evaluated for their antifungal activity against four phytopathogenic fungi (*Alternaria alternata, Alternaria salani, Fusarium oxysporum,* and *Botrytis cinerea*). Most expressed antifungal activity, and the antifungal activity against *A. Salani* and *A. Alternata* was generally better than that against *F. oxysporum* and *B. cinerea.* After screening via the method of the mycelium growth rate and evaluating by EC_50_, the two compounds (**3n** and **4e**) that showed the best antifungal activity against *A. Salani* and *A. Alternata* were taken as potential candidates for the SDH inhibitor.

The promising compound **3n** was selected as the ligand in the docking study. This indicated that the compound was entirely embedded in the cavity, and a conventional hydrogen bond formed between the coumarin moiety and the amino acid residue. TYR58 played an important role in the hydrogen bond. The results of the docking study can be conducive to further exploring the possible fungicidal mechanism, and the interactions between similar fungicidal compounds and SDH.

Furthermore, all these compounds have simple structures, and were easily synthesized. They can act as lead compounds in designing and synthesizing more analogues to screen out an outstanding inhibitor. Further investigations are under way to modify the positions of amino groups or build scaffolds of derivatives in our group.

## 4. Experimental Section

### 4.1. Chemicals and Instruments

Resorcinol, cyanoacetic acid, tetrabutylammonium bromide (TBAB), 1,2-dibromoethane, 1,3-dibromopropane, 1,4-dibromobutane, 1,4-dibromo-2-butene, dibromohexanoic acid, 2-methylvaleric acid, 2-methylhexanoic acid, piperidine hydrochloride, isonipecotic acid, 3-furoic acid, 2-furoic acid, propanedioic acid, 3-hydroxy hexanoic acid, and 3,3-dimethyl allyl bromide were purchased from Sinopharm Co. Ltd., Shanghai, China. ZnCl_2_, NaCl, K_2_CO_3_, and thionyl chloride were purchased from Aladdin Reagent Co. Ltd., Shanghai, China. All other chemicals were commercially available and used without further purification. The progress of the reactions and the purity of products were monitored by TLC using silica gel GF/UV 254. The melting points of synthesized compounds were measured on an X-4 apparatus (uncorrected). ^1^H NMR and ^13^C NMR spectra were detected on a Bruker Avance 400 MHz spectrometer with TMS as an internal standard. Mass spectra (Waters Corp., Massachusetts, USA) were also applied to confirm the synthesized structures.

### 4.2. Synthesis and Characterization

#### 4.2.1. General Procedure of Synthesis

Details of general procedures i~iii as shown in Scheme 1 are provided as follows.

Procedure i [18]: Note that the synthesis of compound **2** is taken as an example here. All manipulations were carried out under nitrogen protection using a vacuum system. The continuous injection of hydrogen chloride and anhydrous ZnCl_2_ was used. The preparation of HCl gas: abundant NaCl was dissolved in 400 mL of HCl solution, and we then added H_2_SO_4_ in drops at room temperature. Resorcinol (22.84 g, 0.2 mol), cyanoacetic acid (19.33 g, 0.2 mol), ZnCl_2_ (13.95 g, 0.1 mmol), and ether (50 mL) were added to a 250 mL three-neck flask fitted with a stirring bar. As the mixture melted, it turned dark yellow. After 5 h of stirring under HCl, a white solid precipitated. At the end of the reaction, ice water was added to quench and refrigerate. A white powdered solid was collected by filtration.

Procedure ii: Note that the synthesis of compound **3a** is taken as an example here. 7-Hydroxy-4-amino coumarin (226.0 mg, 1.25 mmol), anhydrous K_2_CO_3_ (346.9 mg, 2.5 mmol), and tetrabutylammonium bromide (TBAB) (209.1 mg, 0.625 mmol) were dissolved in acetone (10 mL), then the mixture was added to a 50 mL round-bottomed flask fitted with a stirring bar and a condenser at 56 °C. After 30 min, 1,2-dibromoethane (748.4 mg, 4 mmol) was added to the mixture. The reaction was stopped after 23 h. Reprocessing: the mixture was washed with a large amount of ethyl acetate, the filtrate was extracted by saturated NaCl solution 2~3 times, and the organic phase was finally collected.

Procedure iii: Note that the synthesis of compound **3e** is taken as an example here. Dibromohexanoic acid (602.5 mg, 3 mmol) and thionyl chloride (0.6 mL, 7.8 mmol) were added to a 25 mL round-bottomed flask fitted with a stirring bar and a condenser connecting with a gas absorber for SO_2_. When the mixture was heated to 70 °C, it turned brown. The mixture was refluxed for 4 h, then the thionyl chloride was removed under reduced pressure. After adding 7-hydroxy-4-amino coumarin (51.7 mg, 0.3 mmol) and acetone (4 mL), the solution was heated to 56 °C. The reaction was stopped after 24 h.

Procedure i was used for the formation **2** and **2′**. Procedure ii was used for the formation of **3a**, **3b**, **3c**, **3d**, **3h**, **3n**, **4a**, **4b**, **4c**, **4d**, **4h**, and **4n**. Procedure iii was used for the formation of **3e**, **3f**, **3g**, **3i**, **3j**, **3k**, **3l**, **3m**, **3o**, **4e**, **4f**, **4g**, **4i**, **4j**, **4k**, **4l**, **4m**, and **4o**.

#### 4.2.2. Characterization

*7-hydroxy-4-amino coumarin* (**2**): Yield: 8.41 g (23.7%). m.p.: 292.2 °C. ESI-MS m/z for C_9_H_7_NO_3_ [M + H]^+^: 178.05; ^1^H NMR (400 MHz, CD_3_OD_SPE) δ: 7.73 (d, *J* = 8.8 Hz, 1H), 6.78 (dd, *J* = 8.8, 2.3 Hz, 1H), 6.67 (d, *J* = 2.3 Hz, 1H).

*7-hydroxy-4-amino-8-methyl coumarin* (**2′**): 2-methylresorcinol (0.15 mol) was used. Yield: 7.165 g (25%). m.p.: 292.2 °C. ESI-MS m/z for C_10_H_9_NO_3_ [M + H]^+^: 192.06; ^1^H NMR (400 MHz, CD_3_OD_SPE) δ: 7.60 (s, 1H), 6.82 (s, 1H), 5.25 (s, 1H), 2.26 (s, 3H).

*4-amino-7-(2-bromo ethoxy)-2H-chromen-2-one* (**3a**): 1,2-dibromoethane (4 mmol) was used. Yield: 144.2 mg (40.6%). m.p.: 188–190 °C. ESI-MS m/z for C_11_H_10_BrNO_3_ [M + H]^+^: 284.98; ^1^H NMR (400 MHz, CD_3_OD_SPE) δ: 7.83 (d, *J* = 8.9 Hz, 1H), 6.96 (dd, *J* = 8.9, 2.5 Hz, 1H), 6.89 (d, *J* = 2.4 Hz, 1H), 5.28 (s, 1H), 4.49–4.34 (m, 2H), 3.83–3.67 (m, 2H).

*4-amino-7-(3-bromopropoxy)-2H-chromen-2-one* (**3b**): 1,3-dibromopropane (0.75 mmol) was used. Yield: 50.8 mg (68.2%). m.p.: 197.4 °C. ESI-MS m/z: 299.0; [M + H]^+^ C_12_H_12_BrNO_3_. ^1^H NMR (400 MHz, CD_3_OD_SPE) δ 7.81 (d, *J* = 8.9 Hz, 1H), 6.94 (dd, *J* = 8.9, 2.4 Hz, 1H), 6.88 (d, *J* = 2.4 Hz, 1H), 5.27 (s, 1H), 4.21 (t, *J* = 5.9 Hz, 2H), 3.65 (t, *J* = 6.5 Hz, 2H), 2.39–2.28 (m, 2H).

*4-amino-7-(4-bromobutoxy)-2H-chromen-2-one* (**3c**): 1,4-dibromobutane (0.25 mmol) was used. Yield: 45.4 mg (58.2%). m.p.: 211 °C. ESI-MS m/z for C_13_H_14_BrNO_3_ [M + H]^+^: 313.01; ^1^H NMR (400 MHz, CD_3_OD_SPE) δ: 7.79 (d, *J* = 8.9 Hz, 1H), 6.91 (d, *J* = 8.9 Hz, 1H), 6.86 (d, *J* = 2.1 Hz, 1H), 5.26 (s, 1H), 4.11 (t, *J* = 5.9 Hz, 2H), 3.54 (t, *J* = 6.5 Hz, 2H), 2.05 (dd, *J* = 13.7, 6.9 Hz, 2H), 1.98 (dd, *J* = 13.3, 6.7 Hz, 2H).

*(E)-4-amino-7-(4-bromo-2-enoxy) -2H-chromen-2-one* (**3d**): 1,4-dibromo-2-butene (3.75 mmol) was used. Yield: 49.3 mg (21.2%). m.p.: 222.7 °C. ESI-MS m/z for C_13_H_12_BrNO_3_ [M + H]^+^: 311.0; ^1^H NMR (400 MHz, CD_3_OD_SPE) δ: 7.80 (d, *J* = 8.9 Hz, 1H), 6.94 (dd, *J* = 8.9, 2.4 Hz, 1H), 6.87 (d, *J* = 2.3 Hz, 1H), 6.11 (dd, *J* = 15.0, 7.5 Hz, 1H), 6.04 (dd, *J* = 10.1, 5.1 Hz, 1H), 5.26 (s, 1H), 4.67 (d, *J* = 4.9 Hz, 2H), 4.06 (d, *J* = 7.1 Hz, 2H), 3.31 (dd, *J* = 6.1, 4.8 Hz, 2H).

*4-amino-2-oxo-2H-chromen-7-yl-(2-bromohexanoate)* (**3e**): dibromohexanoic acid (3 mmol) was used. Yield: 47.8 mg (45.0%). m.p.: 161–168 °C. ESI-MS m/z for C_15_H_16_BrNO_4_ [M + H]^+^: 355.02; ^1^H NMR (400 MHz, CD_3_OD_SPE) δ: 7.74–7.61 (m, 1H), 6.73–6.41 (m, 1H), 5.45 (s, 1H), 4.30 (t, *J* = 6.6 Hz, 1H), 1.90 (s, 2H), 1.53 (s, 2H), 1.29 (s, 2H), 0.99 (t, *J* = 7.4 Hz, 3H).

*4-amino-2-oxo-2H-chromen-7-yl-(2-methylvalerate)* (**3f**): 2-methylvaleric acid (3 mmol) was used. Yield: 19.8 mg (24.0%). m.p.: 158–165 °C. ESI-MS m/z for C_15_H_17_NO_4_. [M + H]^+^: 276.12; ^1^H NMR (400 MHz, CD_3_OD_SPE) δ: 8.07 (d, *J* = 9.1 Hz, 1H), 6.90 (dd, *J* = 9.1, 2.1 Hz, 1H), 6.68 (d, *J* = 2.1 Hz, 1H), 5.48 (s, 1H), 2.64 (s, 1H), 1.54 (s, 4H), 1.51 (s, 6H).

*4-amino-2-oxochromen-7-yl-(2-methylhexanoate)* (**3g**): 2-methylhexanoic acid (3 mmol) was used. Yield: 41.0 mg (47.3%). m.p.: 168.3 °C. ESI-MS m/z for C_16_H_19_NO_4_ [M + H]^+^: 290.13; ^1^H NMR (400 MHz, CD_3_OD_SPE) δ: 7.90 (d, *J* = 9.2 Hz, 1H), 6.73 (dd, *J* = 9.2, 2.2 Hz, 1H), 6.45 (d, *J* = 2.2 Hz, 1H), 5.45 (s, 1H), 2.62 (s, 1H), 1.90 (s, 2H), 1.53 (s, 4H), 1.51 (s, 3H), 1.34–1.06 (m, 3H). ^13^C NMR (101 MHz, CD_3_OD_SPE) δ 178.16, 165.26, 161.14, 157.55, 153.29, 125.37, 124.24, 122.68, 120.90, 86.38, 38.71, 31.50, 26.28, 24.13, 19.53, 15.18.

*4-amino-7-(piperidin-N-) ethoxy -2H-chromen-2-one* (**3h**): piperidine hydrochloride (0.75 mmol) was used. Yield: 45.1 mg (62.6%). m.p.: 193.4 °C. ESI-MS m/z for C_16_H_20_N_2_O_3_ [M + H]^+^: 289.15; ^1^H NMR (400 MHz, CD_3_OD_SPE) δ: 7.80 (d, *J* = 8.9 Hz, 1H), 6.94 (dd, *J* = 8.9, 2.4 Hz, 1H), 6.87 (d, *J* = 2.3 Hz, 1H), 5.26 (s, 1H), 4.22 (t, *J* = 5.5 Hz, 2H), 2.82 (t, *J* = 5.5 Hz, 2H), 2.57 (s, 4H), 1.64 (dt, *J* = 11.0, 5.6 Hz, 6H).

*4-amino-2-oxochromen-7-yl-piperidine formanoate* (**3i**): isonipecotic acid (18 mmol) was used. Yield: 83.2 mg (1.6%). m.p.: > 235 °C. ESI-MS m/z for C_15_H_16_N_2_O_4_ [M + H]^+^: 289.11; ^1^H NMR (400 MHz, CD_3_OD_SPE) δ: 7.79 (d, *J* = 8.9 Hz, 1H), 6.83 (dd, *J* = 8.8, 2.4 Hz, 1H), 6.69 (d, *J* = 2.3 Hz, 1H), 2.42 (d, *J* = 11.2 Hz, 1H), 2.38–2.13 (m, 4H), 1.29 (s, 4H).

*4-amino-8-methyl-2-oxochromen-7-yl-(3-furancarboxanoate)* (**3j**): 3-furoic acid (0.45 mmol) was used. Yield: 53.8 mg (44.1%). m.p.: 210–222 °C. ESI-MS m/z for C_14_H_9_NO_5_ [M + H]^+^: 272.05; ^1^H NMR (400 MHz, CD_3_OD_SPE) δ: 8.44 (s, 1H), 7.99 (d, *J* = 8.7 Hz, 1H), 7.74–7.68 (m, 1H), 7.28 (d, *J* = 2.1 Hz, 1H), 7.25 (dd, *J* = 8.7, 2.2 Hz, 1H), 6.93 (d, *J* = 1.7 Hz, 1H), 5.40 (s, 1H).

*4-amino-8-methyl-2-oxochromen-7-yl-(2-furancarboxanoate)* (**3k**): 2-furoic acid (0.45 mmol) was used. Yield: 28.3 mg (28.3%). m.p.: 207–210 °C. ESI-MS m/z for C_14_H_9_NO_5_ [M + H]^+^: 272.05; ^1^H NMR (400 MHz, CD_3_OD_SPE) δ: 8.00 (d, *J* = 8.7 Hz, 1H), 7.91 (s, 1H), 7.54 (d, *J* = 3.5 Hz, 1H), 7.31 (d, *J* = 2.1 Hz, 1H), 7.27 (dd, *J* = 8.7, 2.2 Hz, 1H), 6.74 (dd, *J* = 3.4, 1.7 Hz, 1H), 5.40 (s, 1H).

*4-amino-2-oxochromen-7-yl-(monomethyl malonate)* (**3l**): propanedioic acid (3 mmol) was used. Yield: 20.3 mg (23.1%). m.p.: 166–171 °C. ESI-MS m/z for C_13_H_11_NO_6_ [M + H]^+^: 278.06; ^1^H NMR (400 MHz, CD_3_OD_SPE) δ: 8.01 (d, *J* = 9.2 Hz, 1H), 6.84 (dd, *J* = 9.1, 1.9 Hz, 1H), 6.60 (d, *J* = 1.9 Hz, 1H), 5.49 (s, 1H), 3.69 (s, 3H), 2.65 (s, 2H).

*4-amino-2-oxochromen-7-yl-(3-hydroxyhexanoate)* (**3m**): 3-hydroxy hexanoic acid (3.5 mmol) was used. Yield: 15.1 mg (17.3%). m.p.: 127–135 °C. ESI-MS m/z for C_15_H_17_NO_5_ [M + H]^+^: 292.11; ^1^H NMR (400 MHz, CD_3_OD_SPE) δ: 7.89 (d, *J* = 9.2 Hz, 1H), 6.71 (dd, *J* = 9.2, 2.2 Hz, 1H), 6.44 (d, *J* = 2.2 Hz, 1H), 5.43 (s, 1H), 3.82 (s, 1H), 1.88 (s, 2H), 1.51 (s, 2H), 1.27 (s, 2H), 0.89 (t, *J* = 7.1 Hz, 3H).

*4-amino-7-(3-methylbutyl-2-enoxy) 2H-chromen-2-one* (**3n**): 3,3-dimethyl allyl bromide (1.25 mmol) was used. Yield: 19.0 mg (31.0%). m.p.: 248.6 °C. ESI-MS m/z: 246.11; [M + H]^+^ C_14_H_15_NO_3_. ^1^H NMR (400 MHz, CD_3_OD_SPE) δ: 7.78 (d, *J* = 8.8 Hz, 1H), 6.77 (dd, *J* = 8.8, 2.3 Hz, 1H), 6.66 (d, *J* = 2.2 Hz, 1H), 4.93 (s, 2H), 4.83 (s, 2H), 1.80 (s, 6H).

*4-amino-2-oxochromen-7-yl-(2-bromoamanoate)* (**3o**): 2-bromovaleric acid (10 mmol) was used. Yield: 120.3 mg (11.8%). m.p.: 173 °C. ESI-MS m/z for C_14_H_14_BrNO_4_ [M + H]^+^: 341.01; ^13^C NMR (151 MHz, MeOD) δ: 8.18 (d, *J* = 9.1 Hz, 1H), 7.03 (dd, *J* = 9.1, 2.1 Hz, 1H), 6.85 (d, *J* = 2.1 Hz, 1H), 5.53 (s, 1H), 2.68 (s, 2H), 1.29 (s, 2H), 1.02–0.76 (m, 3H). ^13^C NMR (151 MHz, MeOD) δ: 172.85, 161.61, 156.87, 153.77, 126.03, 124.65, 120.72, 120.46, 87.77, 38.61, 31.48, 26.30, 14.96.

*4-amino-7-(2-bromo ethoxy)-8-methyl-2H-chromen-2-one* (**4a**): Yield: 19.6 mg (26.31%). m.p.: 193.2 °C. ESI-MS m/z for C_12_H_12_BrNO_3_ [M + H]^+^: 299.0; ^1^H NMR (400 MHz, CD_3_OD_SPE) δ: 7.74 (s, 1H), 7.01 (s, 1H), 5.30 (s, 1H), 4.46 (s, 2H), 3.79 (s, 2H), 2.32 (s, 3H).

*4-amino-7-(3-bromopropoxy)-8-methyl-2H-chromen-2-one* (**4b**): Yield: 45.4 mg (58.21%). m.p.: 212.4 °C. ESI-MS m/z for C_13_H_14_BrNO_3_ [M + H]^+^: 313.01; ^1^H NMR (400 MHz, CDCl_3_) δ: 7.74 (d, *J* = 8.9 Hz, 1H), 7.01 (d, *J* = 8.9 Hz, 1H), 5.29 (s, 1H), 4.18 (t, *J* = 5.9 Hz, 2H), 3.58 (t, *J* = 6.4 Hz, 2H), 2.29 (s, 3H), 2.07 (dd, *J* = 8, 6.5 Hz, 2H). ^13^C NMR (101 MHz, CDCl_3_) δ: 165.90, 162.80, 155.83, 150.74, 125.31, 120.22, 116.27, 110.85, 95.08, 72.24, 31.75, 29.85.

*4-amino-7-(4-bromobutoxy)-8-methyl-2H-chromen-2-one* (**4c**): Yield: 16.7 mg (20.49%). m.p.: 152 °C. ESI-MS m/z for C_14_H_16_BrNO_3_ [M + H]^+^: 327.03; ^1^H NMR (400 MHz, CD_3_OD_SPE) δ: 7.74 (d, *J* = 8.9 Hz, 1H), 7.01 (d, *J* = 8.9 Hz, 1H), 5.29 (s, 1H), 4.18 (t, *J* = 5.9 Hz, 2H), 3.58 (t, *J* = 6.4 Hz, 2H), 2.29 (s, 3H), 2.11 (dd, *J* = 8, 6.9 Hz, 2H), 2.03 (m, 2H).

*(E)-4-amino-8-methyl-7-(4-bromo-2-enoxy) -2H-chromen-2-one* (**4d**): Yield: 45.67 mg (9.40%). m.p.: 248.6 °C. ESI-MS m/z for C_14_H_14_BrNO_3_ [M + H]^+^: 325.01; ^1^H NMR (400 MHz, CD_3_OD_SPE) δ: 8.53 (s, 1H), 8.09 (d, *J* = 9.1 Hz, 1H), 7.53 (s, 1H), 7.03 (s, 1H), 5.52 (s, 1H), 3.96–3.75 (m, 2H), 3.55 (dd, *J* = 12.7, 6 Hz, 2H), 1.66 (s, 3H).

*4-amino-8-methyl-2-oxochromen-7-yl-(2-bromohexanoate)* (**4e**): Yield: 13.8 mg (7.5%). m.p.: 174–183 °C. ESI-MS m/z for C_16_H_18_BrNO_4_ [M + H]^+^: 369.04; ^1^H NMR (400 MHz, CD_3_OD_SPE) δ: 7.07 (d, *J* = 9.0 Hz, 1H), 6.73–6.66 (m, 1H), 5.51 (s, 1H), 4.22 (s, 1H), 2.27 (s, 3H), 1.96 (t, *J* = 4.7 Hz, 2H), 1.35 (s, 2H), 1.26 (s, 3H), 1.11 (s, 2H). ^13^C NMR (101 MHz, CD_3_OD_SPE) δ: 164.87, 164.00, 162.24, 154.67, 153.92, 125.06, 123.40, 120.52, 116.11, 87.81, 46.79, 31.47, 26.34, 23.19, 14.25.

*4-amino-8-methyl-2-oxochromen-7-yl-(2-methylvaleranoate)* (**4f**): Yield: 21.9 mg (5.05%). m.p.: > 302 °C. ESI-MS m/z for C_16_H_19_NO_4_ [M + H]^+^: 290.13; ^1^H NMR (400 MHz, CD_3_OD_SPE) δ: 7.68 (dd, *J* = 4, 3.4 Hz, 1H), 6.89 (d, *J* = 9.2 Hz, 1H), 5.49 (s, 1H), 2.66 (s, 1H), 1.91 (s, 3H), 1.55 (d, J = 3.5 Hz, 2H), 1.36 (d, *J* = 4.9 Hz, 2H), 1.23 (s, 3H), 0.92 (d, *J* = 7.2 Hz, 3H). ^13^C NMR (101 MHz, CD_3_OD_SPE) δ 171.38, 165.30, 161.37, 152.65, 150.85, 130.49, 129.87, 124.52, 112.66, 88.82, 40.18, 30.74, 24.03, 15.15, 14.39.

*4-amino-8-methyl-2-oxochromen-7-yl-(2-methylhexanoate)* (**4g**): Yield: 12.7 mg (2.65%). m.p.: > 302 °C. ESI-MS m/z for C_17_H_21_NO_4_ [M + H]^+^: 304.15; ^1^H NMR (400 MHz, CD_3_OD_SPE) δ: 8.16 (d, *J* = 8.8 Hz, 1H), 7.10 (d, *J* = 8.8 Hz, 1H), 5.55 (s, 1H), 3.88 (s, 1H), 2.70 (s, 2H), 2.30 (s, 3H), 1.34 (d, *J* = 7.2 Hz, 2H), 1.24 (d, *J* = 6.9 Hz, 2H), 0.94–0.87 (m, 3H). ^13^C NMR (101 MHz, CD_3_OD_SPE) δ: 170.92, 164.74, 162.17, 154.60, 153.97, 125.07, 123.53, 120.52, 116.42, 87.71, 38.51, 31.48, 30.74, 26.34, 15.16, 14.43.

*4-amino-8-methyl-7-(piperidin-N-) ethoxy -2H-chromen-2-one* (**4h**): Yield: 31.0 mg (41.33%). m.p.: 221.6 °C. ESI-MS m/z for C_17_H_22_N_2_O_3_ [M + H]^+^: 303.17; ^1^H NMR (400 MHz, CD_3_OD_SPE) δ: 7.75 (d, *J* = 8.9 Hz, 1H), 7.02 (d, *J* = 9.0 Hz, 1H), 5.29 (s, 1H), 4.30 (t, *J* = 5.5 Hz, 2H), 2.29 (s, 3H), 1.68 (s, 2H), 1.53 (d, *J* = 5.1 Hz, 1H), 1.43 (dd, *J* = 7.36, 7.36 Hz, 2H), 1.28 (d, *J* = 14.6 Hz, 4H).

*4-amino-8-methyl-2-oxochromen-7-yl-piperidine formanoate* (**4i**): Yield: 26.5 mg (4.39%). m.p.: > 302 °C. ESI-MS m/z for C_16_H_18_N_2_O_4_ [M + H]^+^: 592.26; ^1^H NMR (400 MHz, CD_3_OD_SPE) δ: 8.06 (d, *J* = 9.0 Hz, 1H), 7.01 (d, *J* = 9.2 Hz, 1H), 5.52 (s, 1H), 2.68 (s, 1H), 2.27 (s, 3H), 1.58 (s, 4H), 1.56 (s, 4H). ^13^C NMR (101 MHz, CD_3_OD_SPE) δ: 184.35, 171.63, 169.29, 156.58, 155.89, 124.85, 123.25, 117.83, 87.29, 45.11, 38.58, 26.34.

*4-amino-7-(3-furoyloxy)-8-methyl-benzopyran-2-one* (**4j**): Yield: 20.8 mg (3.65%). m.p.: 220 °C. ESI-MS m/z for C_15_H_11_NO_5_ [M + H]^+^: 286.07; ^1^H NMR (400 MHz, CD_3_OD_SPE) δ: 8.09 (s, 1H), 7.81 (d, *J* = 8.8 Hz, 1H), 7.55 (s, 1H), 6.92 (s, 1H), 6.72 (s, 1H), 5.39 (s, 1H), 2.27 (s, 3H). ^13^C NMR (101 MHz, CD_3_OD_SPE) δ: 166.44, 162.13, 154.27, 153.15, 150.92, 149.33, 146.22, 145.37, 121.62, 120.69, 120.39, 119.28, 110.79, 84.36.

*4-amino-7-(2-furoyloxy)-8-methyl-benzopyran-2-one* (**4k**): Yield: 18.5 mg (25.96%). m.p.: 212 °C. ESI-MS m/z for C_15_H_11_NO_5_ [M + H]^+^: 286.07; ^1^H NMR (400 MHz, CD_3_OD_SPE) δ: 7.82 (d, *J* = 8.9 Hz, 1H), 7.54 (d, *J* = 3.6 Hz, 1H), 7.19 (d, *J* = 8.8 Hz, 1H), 6.73 (d, *J* = 1.7 Hz, 1H), 6.27 (d, *J* = 8.1 Hz, 1H), 5.39 (s, 1H), 2.28 (s, 3H). ^13^C NMR (101 MHz, CD_3_OD_SPE) δ: 166.44, 158.70, 157.49, 154.17, 152.86, 149.68, 146.05, 121.68, 121.47, 120.77, 119.55, 118.60, 113.66, 84.44.

*4-amino-8-methyl-2-oxochromen-7-yl-(monomethyl malonate)* (**4l**): Yield: 21.1 mg (1.94%). m.p.: > 302 °C. ESI-MS m/z for C_14_H_13_NO_6_ [M + H]^+^: 292.08; ^1^H NMR (400 MHz, CD_3_OD_SPE) δ: 7.64 (dd, *J* = 9.24, 9.1 Hz, 1H), 6.79–6.58 (m, 1H), 5.38 (s, 1H), 3.78 (d, *J* = 9.3 Hz, 3H), 3.48 (q, *J* = 7.0 Hz, 2H), 2.12 (d, *J* = 19.2 Hz, 3H). ^13^C NMR (101 MHz, CD_3_OD_SPE) δ: 170.41, 168.67, 165.96, 161.17, 154.17, 150.37, 122.79, 118.09, 86.10, 56.84, 31.53.

*4-amino-8-methyl-2-oxochromen-7-yl-(3-hydroxyhexanoate)* (**4m**): Yield: 73.9 mg (16.15%). m.p.: 236–244 °C. ESI-MS m/z for C_16_H_19_NO_5_ [M + H]^+^: 306.13; ^1^H NMR (400 MHz, CD_3_OD_SPE) δ: 7.56 (d, *J* = 8.8 Hz, 1H), 6.74 (d, *J* = 8.8 Hz, 1H), 5.34 (s, 1H), 3.65 (s, 1H), 2.22 (s, 3H), 2.18–2.13 (m, 2H), 1.39 (s, 2H), 1.36 (d, J = 6.7 Hz, 2H), 1.21 (s, 3H).

*4-amino-8-methyl-7-(3-methylbutyl-2-enoxy) 2H-chromen-2-one* (**4n**): Yield: 29.9 mg (11.54%). m.p.: 220 °C. ESI-MS m/z for C_15_H_17_NO_3_ [M + H]^+^: 260.12; ^1^H NMR (400 MHz, CD_3_OD_SPE) δ: 7.64 (d, *J* = 8.8 Hz, 1H), 6.80 (d, *J* = 8.8 Hz, 1H), 5.11 (s, 1H), 4.31 (s, 1H), 2.26 (s, 3H), 1.80 (d, *J* = 8.2 Hz, 6H). ^13^C NMR (101 MHz, CD_3_OD_SPE) δ: 165.90, 159.74, 151.29, 147.39, 132.35, 129.87, 120.91, 120.54, 114.31, 112.40, 89.12, 66.66, 24.95, 20.26. ^13^C NMR (101 MHz, CD_3_OD_SPE) δ 165.90, 159.74, 151.29, 147.39, 132.35, 129.87, 120.91, 120.54, 114.31, 112.40), 89.12, 66.66, 24.95, 20.26.

*4-amino-8-methyl-2-oxochromen-7-yl-(2-bromoamanoate)* (**4o**): Yield: 243.2 mg (34.35%). m.p.: 245.2 °C. ESI-MS m/z for C_15_H_16_BrNO_4_ [M + H]^+^: 355.02; ^1^H NMR (600 MHz, CD_3_OD) δ: 8.08 (d, *J* = 9.0 Hz, 1H), 7.09 (d, *J* = 9.0 Hz, 1H), 5.53 (s, 1H), 2.30 (s, 3H), 2.21 (d, *J* = 4.9 Hz, 2H), 1.42 (dd, *J* = 12.48, 12.6 Hz, 3H), 1.28 (s, 2H). ^13^C NMR (151 MHz, CD_3_OD) δ: 163.99, 162.26, 154.73, 154.68, 153.93, 125.05, 123.35, 120.55, 116.08, 87.83, 38.47, 31.47, 26.34, 8.19, 3.24.

### 4.3. Procedure of Antifungal Activity

The in vitro antifungal activity of compounds **3a**–**3o** and **4a**–**4o** against *Alternaria alternata, Alternaria salani, Fusarium oxysporum,* and *Botrytis cinerea* was assessed by the mycelium growth rate method [6,7]. The tested fungi were supplied by Lanzhou Institute of Chemical Physics, Chinese Academy of Sciences. The synthesized compounds were dissolved in a 20% DMSO aqueous solution. The solution of each compound was added to sterilized potato dextrose agar to produce a final concentration of 200 µg/mL for the following antifungal test. After the mycelium of the fungi was transferred to the test plate and incubated at 25 °C over a certain period, the diameter of each strain was measured. Each experiment was performed three times, and the data were averaged. The percentage inhibition was calculated as follows: *I* = (*B* − *A*)/(*B* − 0.7) × 100%, where *I* is the percentage of inhibition, *A* is the average mycelia diameter (mm) with the compounds in Petri dishes, *B* is the average mycelia diameter in the blank Petri dishes, and the value of 0.7 mm is the hyphal diameter.

### 4.4. Docking Studies and Results Visualization

The most promising inhibitor, **3n,** was docked into the active UQ-site of SDH (PDB code: 1YQ3) [29] using the open-source Autodock Vina code [28]. Docked positions in the active site were visualized, and the analysis of docking results was performed using the PyMOL program [30]. Gasteiger charges were added to atoms. Grid points for the search space were set to 15 × 15 × 15 Å^3^ around the active center. The empirical free energy function and Lamarckian genetic algorithm were applied. The scoring function included the van der Waals interaction, the hydrogen bonding, and the Coulombic electrostatic potential.

## Figures and Tables

**Table 1:** Antifungal activity of synthesized compounds without 8-methyl at 200 μg/mL in 96 h.

Compd.	The Inhibition Rate (96 h) (%; Mean ± SD; N = 3)			
	*B. Cinerea*	*A. Salani*	*F. Oxysporum*	*A. Alternata*
**2**	-	1.70 ± 2.4	-	3.10 ± 2.6
**3a**	18.40 ± 2.2	33.10 ± 1.2	15.40 ± 2.5	49.60 ± 1.0
**3b**	5.50 ± 3.4	19.40 ± 0.9	8.20 ± 1.8	17.60 ± 2.5
**3c**	0.00 ± 1.9	20.00 ± 1.0	-	31.10 ± 2.5
**3d**	23.90 ± 2.2	13.70 ± 1.2	10.60 ± 0.6	24.40 ± 1.8
**3e**	10.00 ± 3.4	12.62 ± 1.6	10.86 ± 3.8	22.64 ± 2.9
**3f**	20.65 ± 3.1	37.22 ± 2.3	16.10 ± 1.6	23.90 ± 1.9
**3g**	5.81 ± 1.0	4.21 ± 3.1	7.12 ± 2.4	2.52 ± 4.0
**3h**	0.06 ± 12.6	0.21 ± 5.7	-	0.02 ± 6.6
**3i**	14.84 ± 0.4	19.09 ± 2.0	13.86 ± 0.8	25.79 ± 4.2
**3j**	0.00 ± 1.7	3.56 ± 1.6	-	0.63 ± 3.1
**3k**	7.10 ± 1.6	6.15 ± 0.6	10.11 ± 3.8	6.29 ± 1.3
**3l**	6.45 ± 2.6	-	2.62 ± 2.7	8.81 ± 0.6
**3m**	9.68 ± 1.5	22.98 ± 1.0	-	8.18 ± 3.1
**3n**	44.80 ± 0.6	76.00 ± 2.5	40.20 ± 2.0	73.70 ± 3.4
**3o**	38.68 ± 0.1	28.70 ± 0.1	27.14 ± 0.1	23.68 ± 0.1
Carbendazim	91.88 ± 0.0	15.16 ± 0.2	86.81 ± 0.0	14.67 ± 0.2
Chlorothalonil	78.36 ± 0.0	58.25 ± 0.1	66.85 ± 0.0	56.69 ± 0.1

“-” represents negative growth.

**Table 2:** Antifungal activity of synthesized compounds with 8-methyl at 200 μg/mL in 96 h.

Compd.	The Inhibition Rate (96 h) (%; Mean ± SD; N = 3)			
	*B. Cinerea*	*A. Salani*	*F. Oxysporum*	*A. Alternata*
**2′**	2.00 ± 3.8	-	12.80 ± 3.4	22.88 ± 3.3
**4a**	7.20 ± 2.4	4.00 ± 0.9	2.42 ± 0.7	19.75 ± 3.8
**4b**	7.74 ± 2.8	-	-	1.80 ± 8.3
**4c**	-	15.69 ± 1.2	5.19 ± 1.7	27.90 ± 3.0
**4d**	2.62 ± 9.2	21.51 ± 3.2	6.71 ± 4.2	16.37 ± 3.0
**4e**	38.34 ± 4.3	45.00 ± 4.9	38.00 ± 0.7	49.28 ± 4.3
**4f**	16.70 ± 1.8	27.60 ± 2.5	15.11 ± 5.1	0.95 ± 1.9
**4g**	22.70 ± 2.8	40.59 ± 2.7	28.33 ± 8.0	44.06 ± 8.3
**4h**	-	4.00 ± 1.0	13.49 ± 1.9	4.08 ± 2.2
**4i**	32.00 ± 0.7	35.31 ± 3.9	5.47 ± 1.6	10.48 ± 1.5
**4j**	14.40 ± 2.8	-	5.88 ± 1.4	12.85 ± 1.8
**4k**	1.60 ± 1.7	3.38 ± 0.9	5.19 ± 1.4	12.85 ± 1.7
**4l**	6.19 ± 4.3	6.49 ± 4.9	2.86 ± 0.7	17.12 ± 4.3
**4m**	7.38 ± 1.8	38.43 ± 2.4	21.21 ± 12.1	21.56 ± 2.6
**4n**	16.00 ± 1.6	45.85 ± 0.9	21.11 ± 1.8	44.20 ± 2.1
**4o**	8.04 ± 0.2	27.29 ± 0.0	20.71 ± 0.0	30.22 ± 0.0
Carbendazim	91.88 ± 0.0	15.16 ± 0.2	86.81 ± 0.0	14.67 ± 0.2
Chlorothalonil	78.36 ± 0.0	58.25 ± 0.1	66.85 ± 0.0	56.69 ± 0.1

“-” represents negative growth.

**Table 3:** In vitro EC_50_ values of selected compounds against *A. Alternata* and *A. Salani* at 200, 100, 50, 25, and 12.5 μg/mL.

Compd.	EC_50_ (μg/mL)	
	*A. Alternata*	*A. Salani*
**3n**	107.4	96.7
**3e**	>500	>500
**4n**	492.4	474
**4e**	144.5	92.1
**3g**	>500	>500
**4g**	436.9	>500

## Data Availability

The data presented in this study are available in article and Supplementary Materials.

## Supplementary Materials

The following supporting information can be downloaded at: https://www.mdpi.com/article/10.3390/molecules27092738/s1.

## Sample Availability

Samples of the compounds are available from the authors.
