# Supplementary material for: Design, Synthesis, and Antifungal Activity of 4-Amino Coumarin Based Derivatives
Source: Molecules. 2022 Apr 24;27(9):2738. doi: 10.3390/molecules27092738 (PMC9104767; doi:10.3390/molecules27092738)
Supplement: Supplementary file 1 [file molecules-27-02738-s001.zip › molecules-1611962-supplementary.pdf]

Supplementary data

Design, synthesis, and antifungal activity of 4-amino coumarin based derivatives

Lu Xu, Jinmeng Yu, Lu Jin*, Le Pan*

College of Chemistry and Chemical Engineering, Xinjiang Agricultural University, Urumqi 830052, China

Table S1. Antifungal activity of synthesized compounds without 8-methyl at 200 μg/mL in 48h.

Compd.	The inhibition rate (48h) (%; mean±SD; N=3)			
	B.Cinerea	*A.Salani*	*F.Oxysporum*	*A.Alternata*
**2**	2.80±1.3	-	0.00±1.2	-
**3a**	23.60±1.8	23.80±0.6	14.30±1.8	51.50±1.6
**3b**	12.50±2.6	14.30±1.6	11.40±1.4	20.60±2.1
**3c**	20.80±1.6	10.20±1.5	5.70±0.8	22.64±1.6
**3d**	44.40±2.6	14.30±0.8	10.00±0.7	13.20±1.4
**3e**	9.40±3.0	26.63±1.8	15.52±2.4	34.50±2.1
**3f**	26.09±2.7	47.93±1.6	20.69±1.3	30.99±1.4
**3g**	2.90±1.2	19.53±2.8	-	15.79±2.8
**3h**	0.02±3.4	0.03±3.1	0.01±2.4	0.05±3.0
**3i**	23.19±0.8	28.99±1.5	20.69±1.2	39.18±3.6
**3j**	4.35±1.3	8.87±1.0	5.17±3.6	0.58±2.0
**3k**	-	17.16±0.4	18.97±2.2	9.94±1.4
**3l**	4.32±1.8	11.24±1.2	3.45±2.0	9.98±1.2
**3m**	-	14.79±1.7	1.72±1.6	-
**3n**	44.40±0.5	78.20±1.6	40.00±1.4	79.40±2.0
**3o**	21.96±0.0	35.18±0.1	26.27±0.0	7.17±0.1
Carbendazim	90.29±0.0	8.13±0.0	92.13±0.1	20.36±0.1
Chlorothalonil	80.39±0.0	66.91±0.1	70.48±0.0	65.24±0.0

“-”represents negative growth.

Table S2. Antifungal activity of synthesized compounds with 8-methyl at 200 μg/mL in 48h.

Compd.	The inhibition rate (48h) (%; mean±SD; N=3)			
	B.Cinerea	*A.Salani*	*F.Oxysporum*	*A.Alternata*
**2'**	15.29±2.8	-	20.00±3.1	0.00±2.0
**4a**	27.06±2.1	3.03±0.6	5.89±0.3	15.07±3.1
**4b**	13.61±2.3	3.00±2.6	-	18.58±7.6
**4c**	12.94±1.1	6.06±0.9	8.24±1.2	38.36±2.6
**4d**	11.57±8.6	22.16±2.9	1.59±3.2	20.48±3.0
**4e**	45.38±4.1	52.66±3.9	54.31±0.2	65.34±4.2
**4f**	1.03±1.6	20.51±2.1	17.91±4.3	18.13±1.4
**4g**	22.31±2.3	47.93±2.6	38.79±7.6	55.68±8.1
**4h**	8.24±0.5	0.00±1.2	22.35±1.3	10.96±2.1
**4i**	-	3.85±2.8	8.96±1.5	-
**4j**	17.65±2.3	1.52±2.8	5.88±1.6	41.10±1.7
**4k**	24.71±1.2	-	17.65±1.7	21.92±1.2
**4l**	10.20±4.0	11.30±4.1	2.20±0.6	28.96±4.3
**4m**	22.22±1.2	36.93±2.6	25.40±7.3	30.52±2.1
**4n**	5.88±1.5	42.42±0.7	12.94±1.6	52.05±1.8
**4o**	13.09±0.0	29.25±0.0	3.73±0.0	38.49±0.0
Carbendazim	90.29±0.0	8.13±0.0	92.13±0.1	20.36±0.1
Chlorothalonil	80.39±0.0	66.91±0.1	70.48±0.0	65.24±0.0

“-”represents negative growth.

Table S3. Antifungal activity of synthesized compounds without 8-methyl at 200 μg/mL in 72h.

Compd.	The inhibition rate (72h) (%; mean±SD; N=3)			
	B.Cinerea	*A.Salani*	*F.Oxysporum*	*A.Alternata*
**2**	0.00±1.2	-	4.50±1.1	-
**3a**	25.20±1.6	32.50±0.8	18.20±1.9	47.30±1.6
**3b**	9.60±2.4	20.50±1.5	9.10±1.6	21.80±2.3
**3c**	4.30±1.5	19.70±1.4	8.20±1.5	23.50±1.2
**3d**	31.30±2.0	15.40±1.5	15.50±1.2	21.80±1.6
**3e**	14.16±2.4	19.83±1.2	14.44±2.4	28.46±2.4
**3f**	23.29±2.8	42.15±1.8	27.78±1.2	27.64±1.4
**3g**	3.20±1.6	16.53±2.6	6.67±2.1	12.20±1.2
**3h**	0.04±3.1	0.12±2.4	0.07±2.6	0.04±2.3
**3i**	21.46±0.6	25.62±2.1	22.22±0.6	32.52±2.1
**3j**	3.20±2.2	1.65±1.2	13.33±3.3	3.25±2.5
**3k**	4.11±1.8	12.40±1.0	17.78±3.2	8.94±1.0
**3l**	7.76±1.4	9.09±2.1	3.33±2.1	13.01±0.8
**3m**	4.11±1.6	17.36±1.4	4.44±2.4	2.44±2.3
**3n**	47.80±0.8	76.90±1.5	39.10±1.6	75.30±2.1
**3o**	38.32±0.0	38.54±0.1	39.86±0.1	23.09±0.1
Carbendazim	91.68±0.0	5.41±0.1	82.63±0.0	14.35±0.1
Chlorothalonil	85.20±0.0	55.02±0.1	74.25±0.1	59.26±0.0

"-"represents negative growth.

Table S4. Antifungal activity of synthesized compounds with 8-methyl at 200 μg/mL in 72h.

Compd.	The inhibition rate (72h) (%; mean±SD; N=3)			
	B.Cinerea	*A.Salani*	*F.Oxysporum*	*A.Alternata*
**2’**	1.16±2.1	-	9.47±3.1	8.64±2.9
**4a**	2.33±2.1	4.60±0.4	6.48±0.3	14.40±3.0
**4b**	5.15±2.3	1.09±2.2	-	8.00±7.6
**4c**	-	21.34±0.9	2.11±1.4	29.22±2.4
**4d**	6.19±9.1	19.43±3.1	3.91±4.2	17.36±2.9
**4e**	40.72±4.1	49.63±3.6	43.75±0.2	59.57±3.8
**4f**	12.87±1.3	30.56±2.3	19.05±4.6	9.45±1.5
**4g**	23.52±2.3	42.96±2.5	32.21±8.1	50.82±7.6
**4h**	-	3.77±0.9	12.36±1.2	6.17±2.4
**4i**	25.74±0.6	30.47±3.1	4.76±1.4	-
**4j**	5.81±2.4	7.12±2.2	6.32±1.3	5.35±1.6
**4k**	-	7.11±0.6	7.37±1.1	12.76±1.3
**4l**	4.29±4.1	9.09±4.5	5.24±0.8	19.64±4.2
**4m**	16.22±1.8	39.37±2.4	26.45±6.8	27.5±2.6
**4n**	12.79±1.2	45.61±0.5	12.63±1.3	47.33±1.8
**4o**	11.76±0.1	29.36±0.1	21.31±0.1	33.57±0.0
Carbendazim	91.68±0.0	5.41±0.1	82.63±0.0	14.35±0.1
Chlorothalonil	85.20±0.0	55.02±0.1	74.25±0.1	59.26±0.0

“-”represents negative growth.

Table S5. Calculated $\log P_{O/W}$ and $\text{Clog}P_{O/W}$ for the lipophilicity.

	$\log P_{O/W}$ [a]	$\text{Clog}P_{O/W}$ [b]		$\log P_{O/W}$ [a]	$\text{Clog}P_{O/W}$ [b]
**3a**	1.99	1.99	**4a**	2.33	2.49
**3b**	2.32	2.32	**4b**	2.64	2.82
**3c**	2.65	2.70	**4c**	2.95	3.20
**3d**	2.46	2.42	**4d**	2.75	2.92
**3e**	3.03	3.06	**4e**	3.33	3.56
**3f**	2.57	2.59	**4f**	2.89	3.09
**3g**	2.89	3.12	**4g**	3.23	3.62
**3h**	2.08	2.60	**4h**	2.40	3.09
**3i**	1.35	0.76	**4i**	1.69	1.26
**3j**	1.77	2.00	**4j**	2.08	2.50
**3k**	1.81	2.00	**4k**	2.13	2.50
**3l**	1.07	0.49	**4l**	1.33	0.99
**3m**	1.77	1.33	**4m**	2.07	1.83
**3n**	2.44	2.96	**4n**	2.77	3.46
**3o**	2.67	2.53	**4o**	2.97	3.03

a. Calculated by SwissADME web tool. Daina, A.; Michielin, O.; Zoete, V. *Sci. Rep.* 2017, *7*, 42717.

b. Calculated by Chemdraw program (Version 16).

^{1}H NMR and ^{13}C NMR

Note that the compounds would not be completely purified and several spectra showed the residual solvent was not thoroughly evaporated.

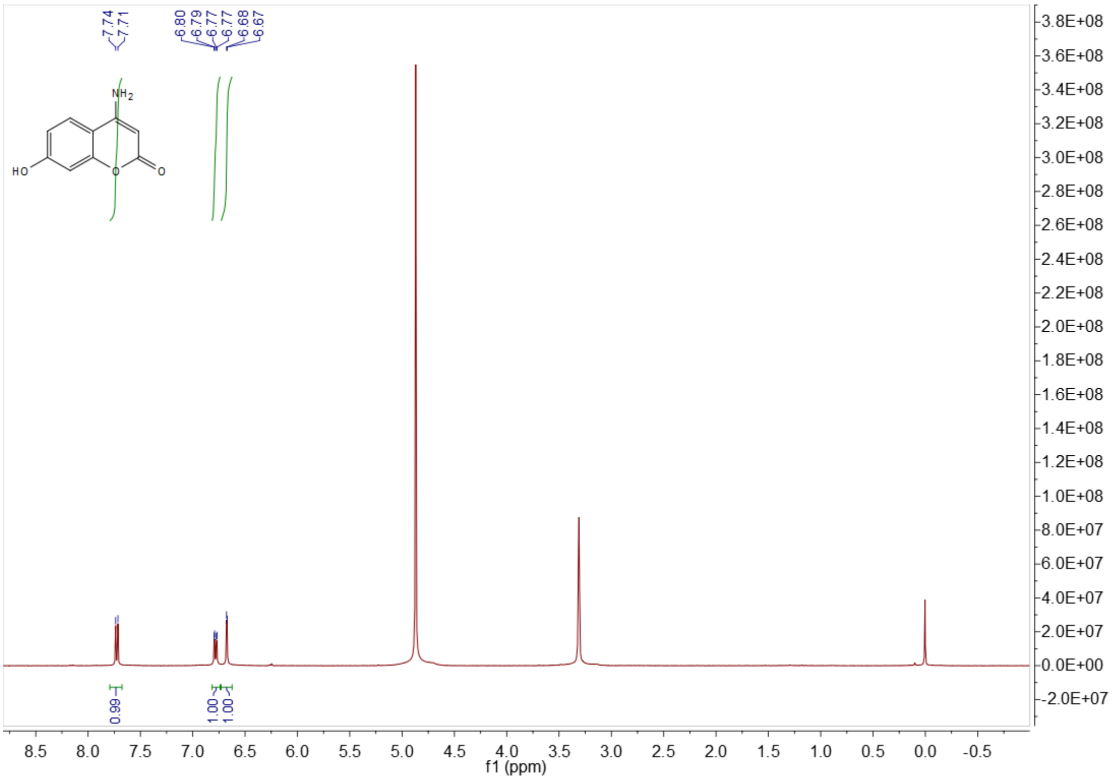

Compound 2 ^{1}H NMR

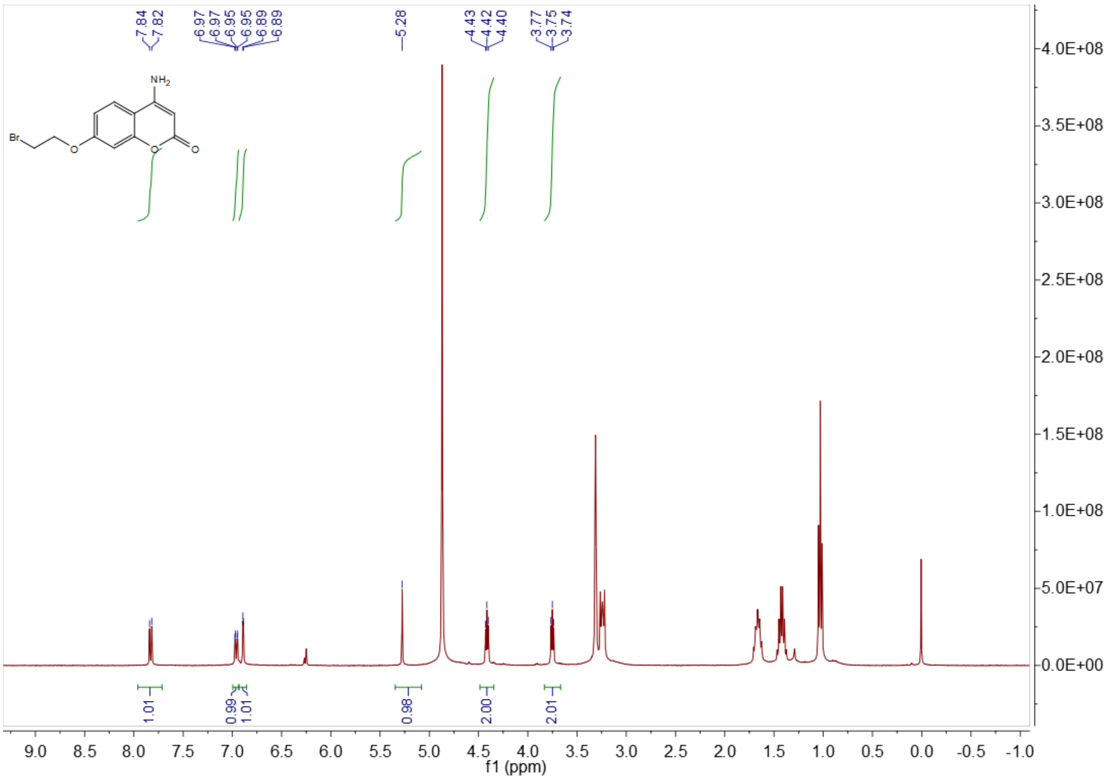

Compound 3a ^{1}H NMR

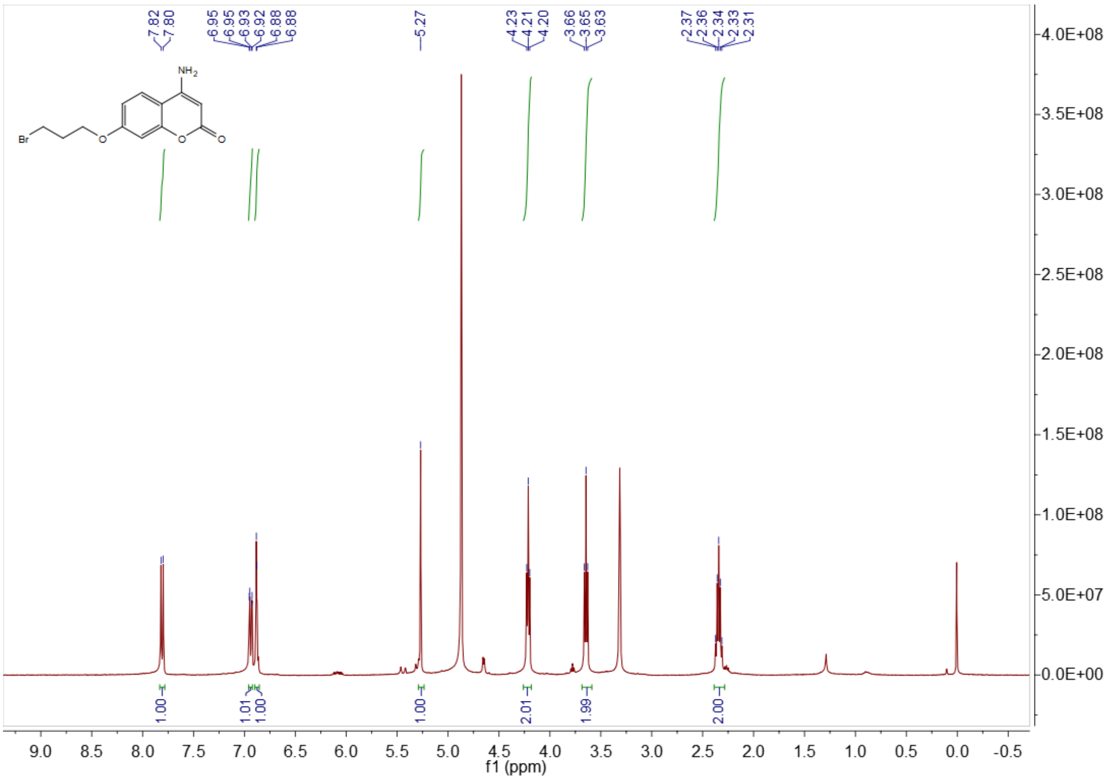

Compound 3b ^{1}H NMR

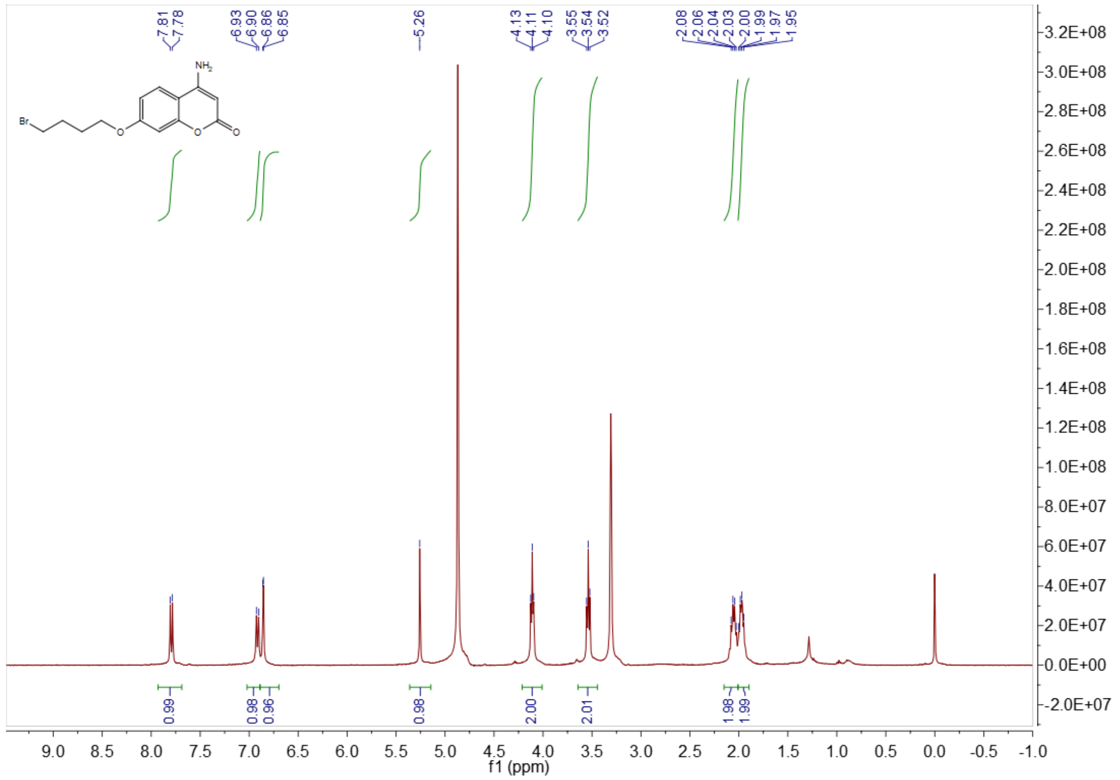

Compound 3c ^{1}H NMR

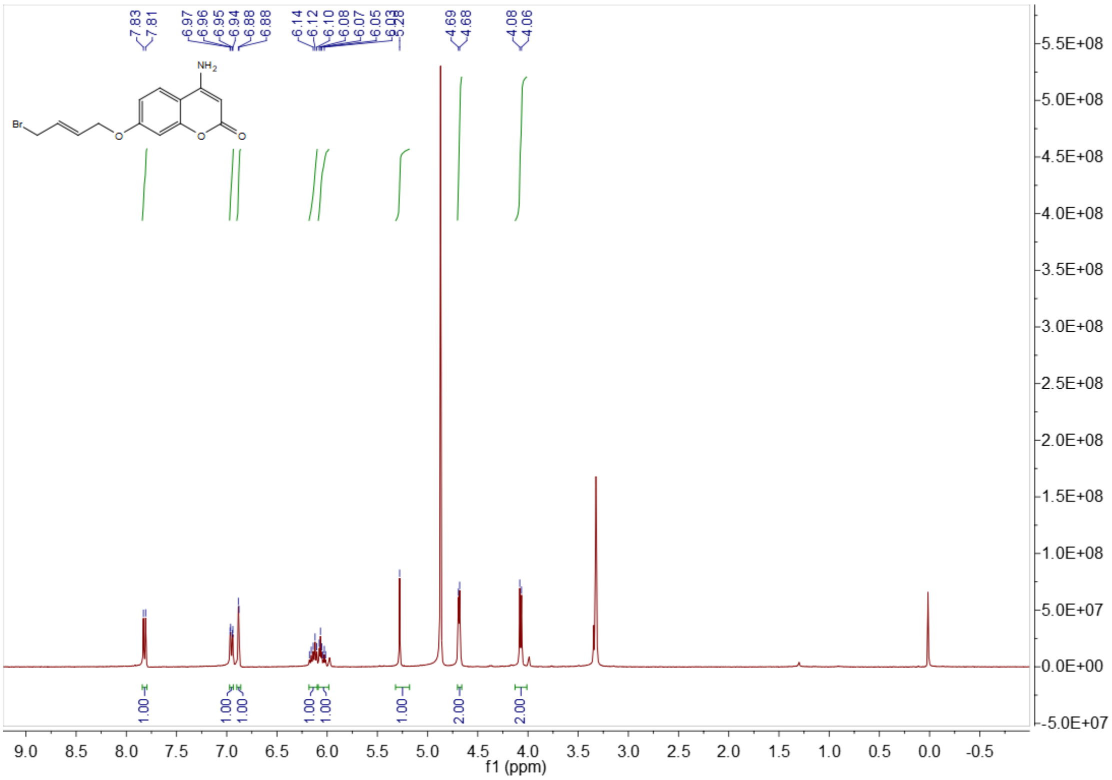

Compound 3d ^{1}H NMR

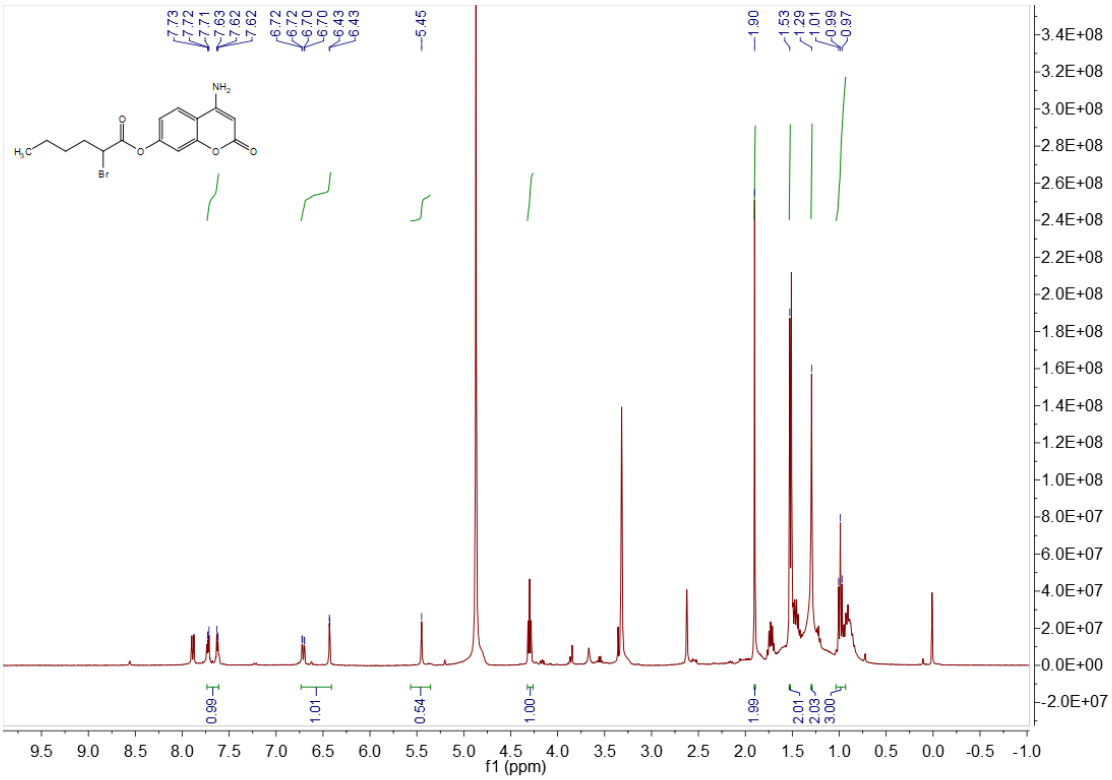

Compound 3e ^{1}H NMR

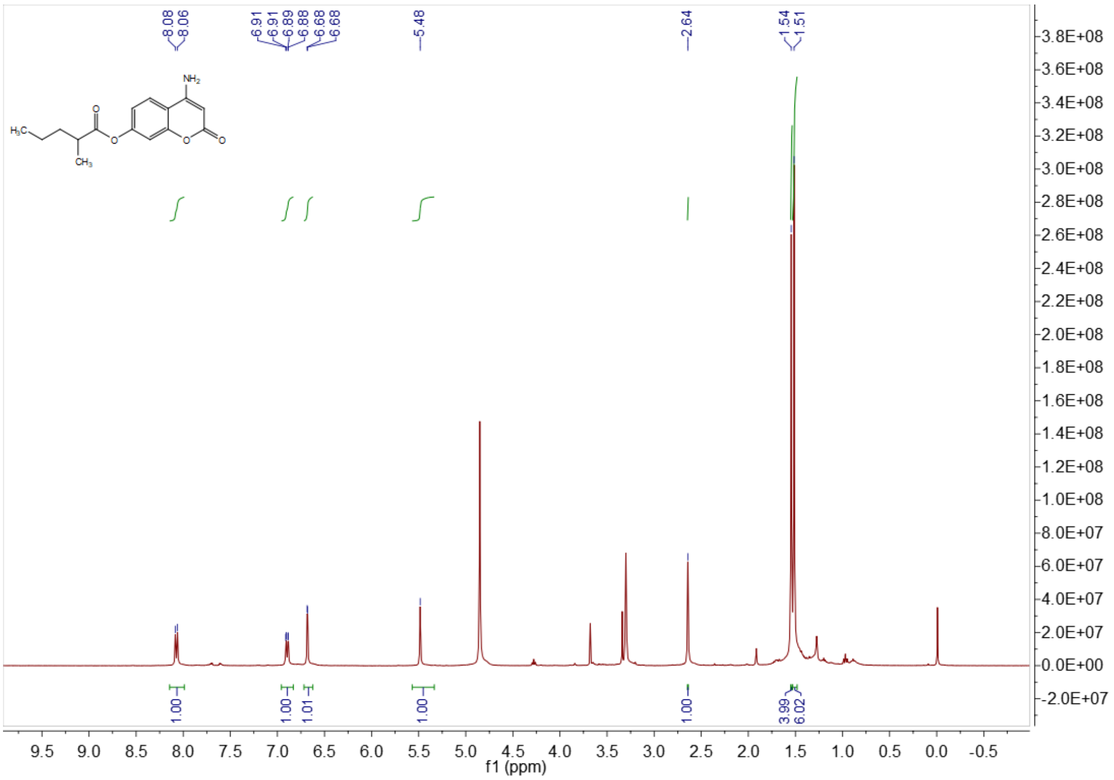

Compound 3f ^{1}H NMR

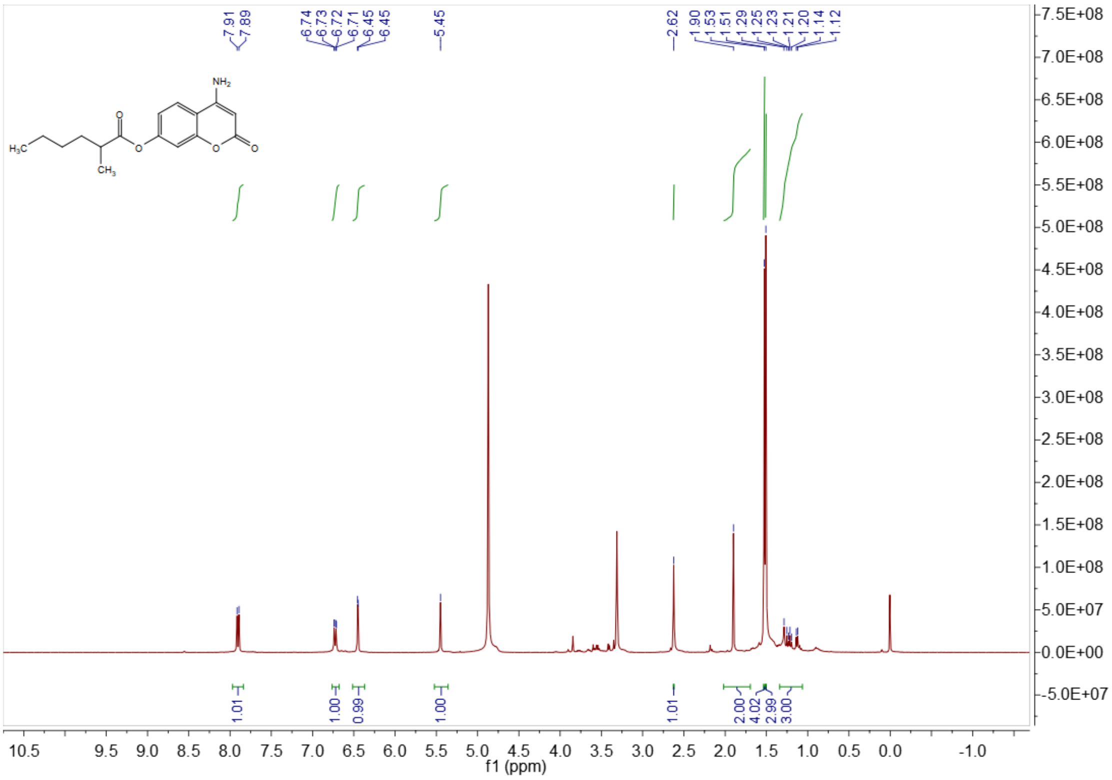

Compound 3g ^{1}H NMR

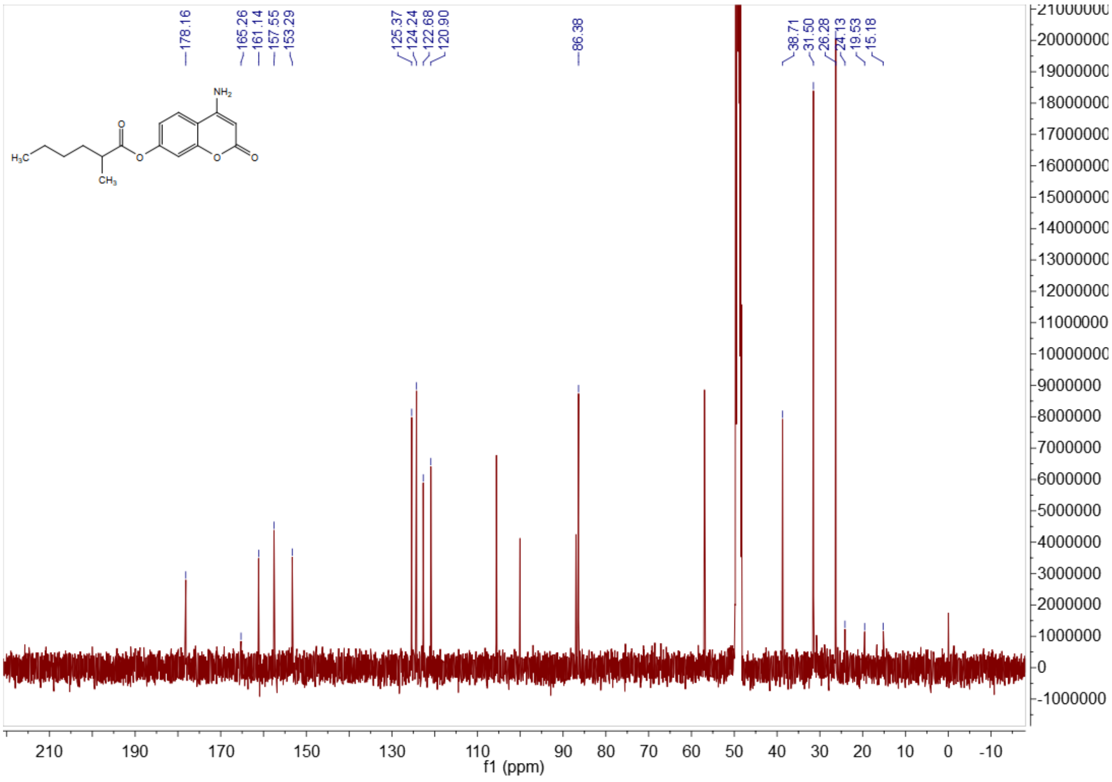

Compound 3g ^{13}C NMR

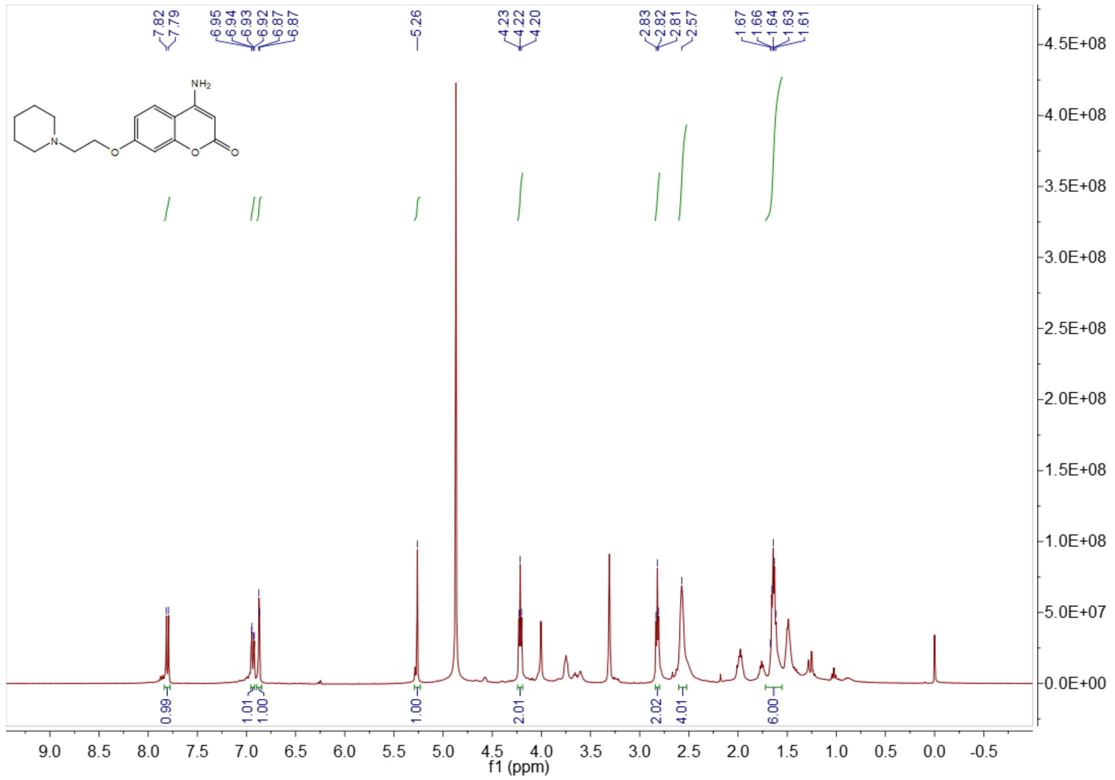

Compound 3h ^{1}H NMR

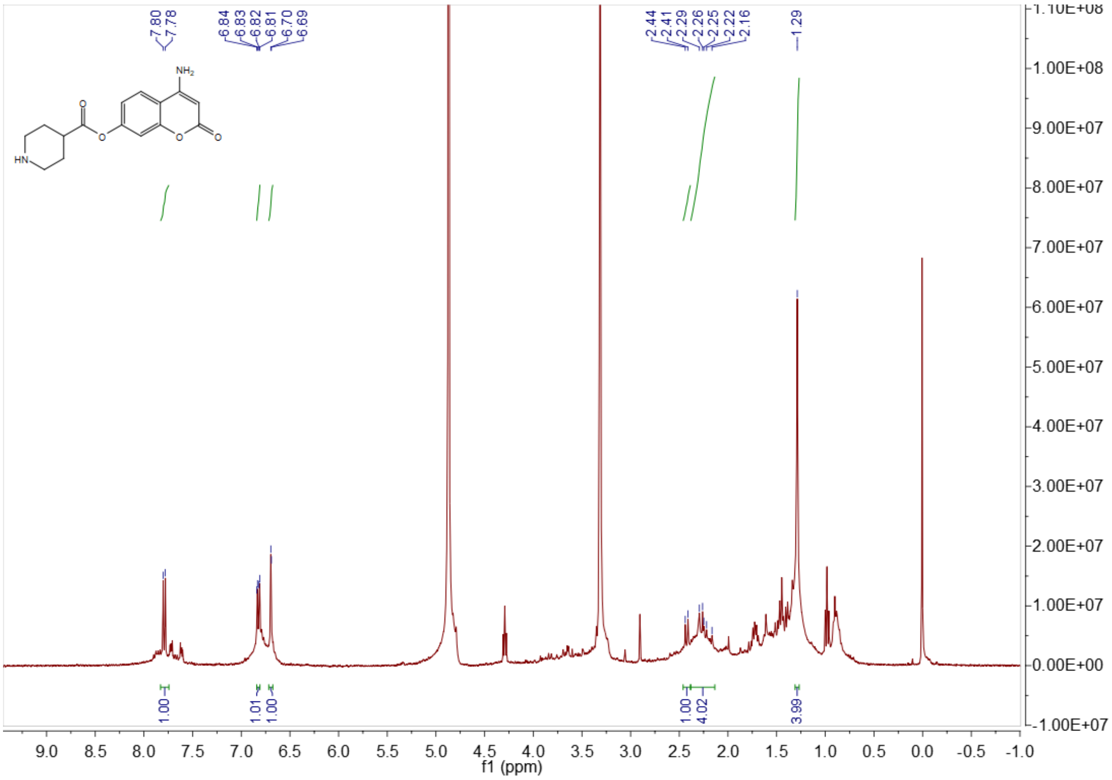

Compound 3i ^{1}H NMR

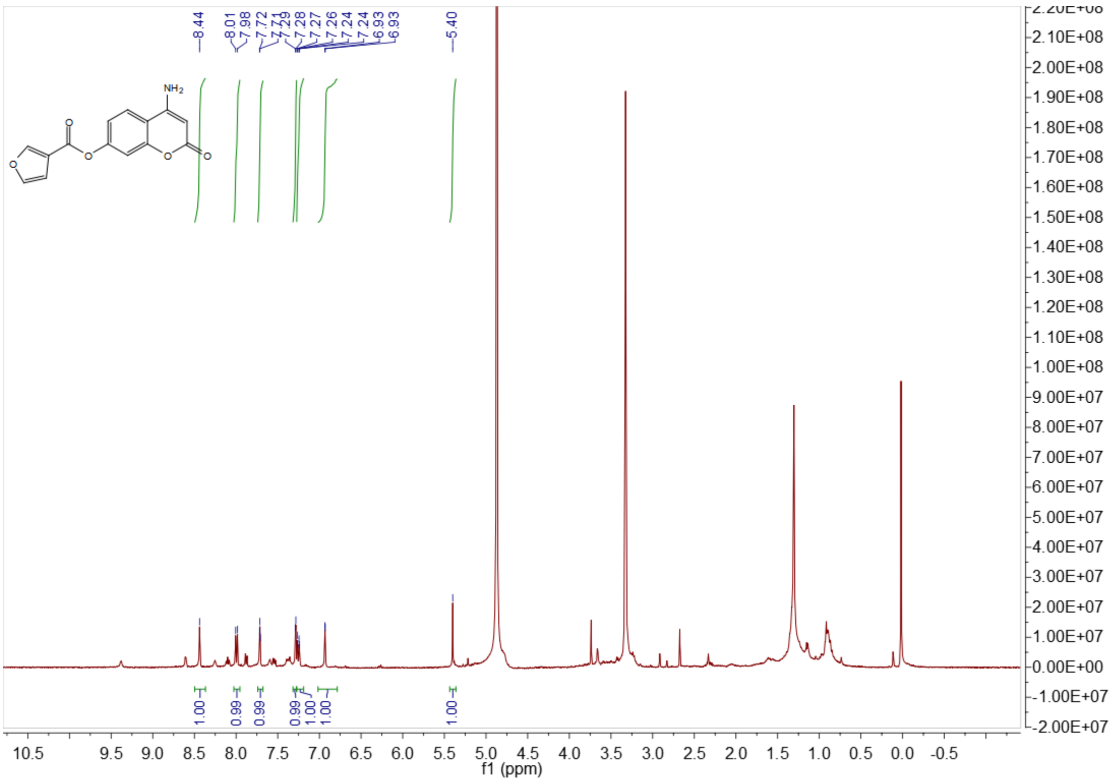

Compound 3j ^{1}H NMR

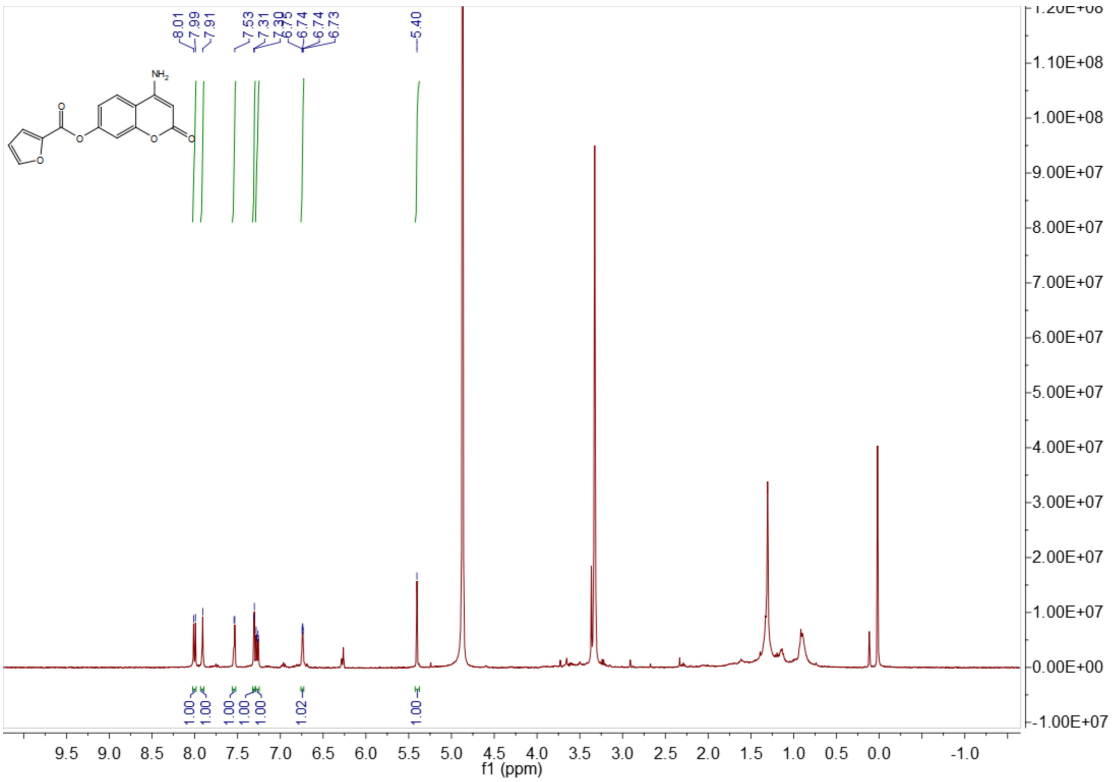

Compound 3k ^{1}H NMR

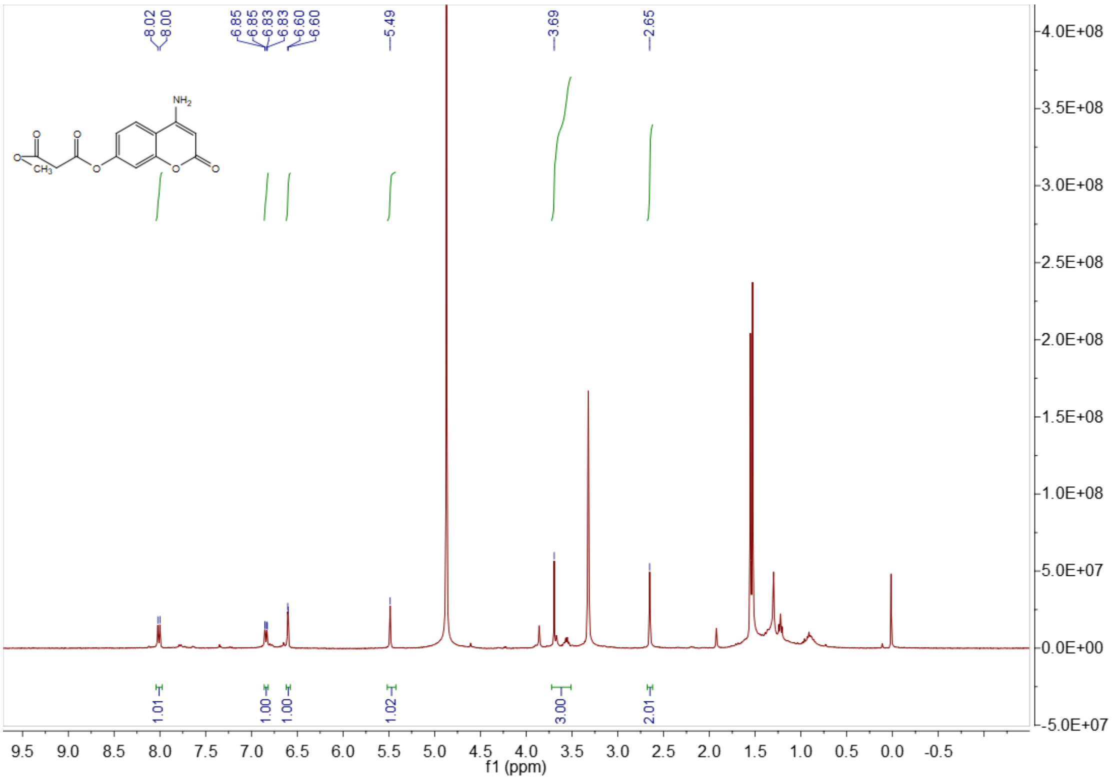

Compound 3l ^{1}H NMR

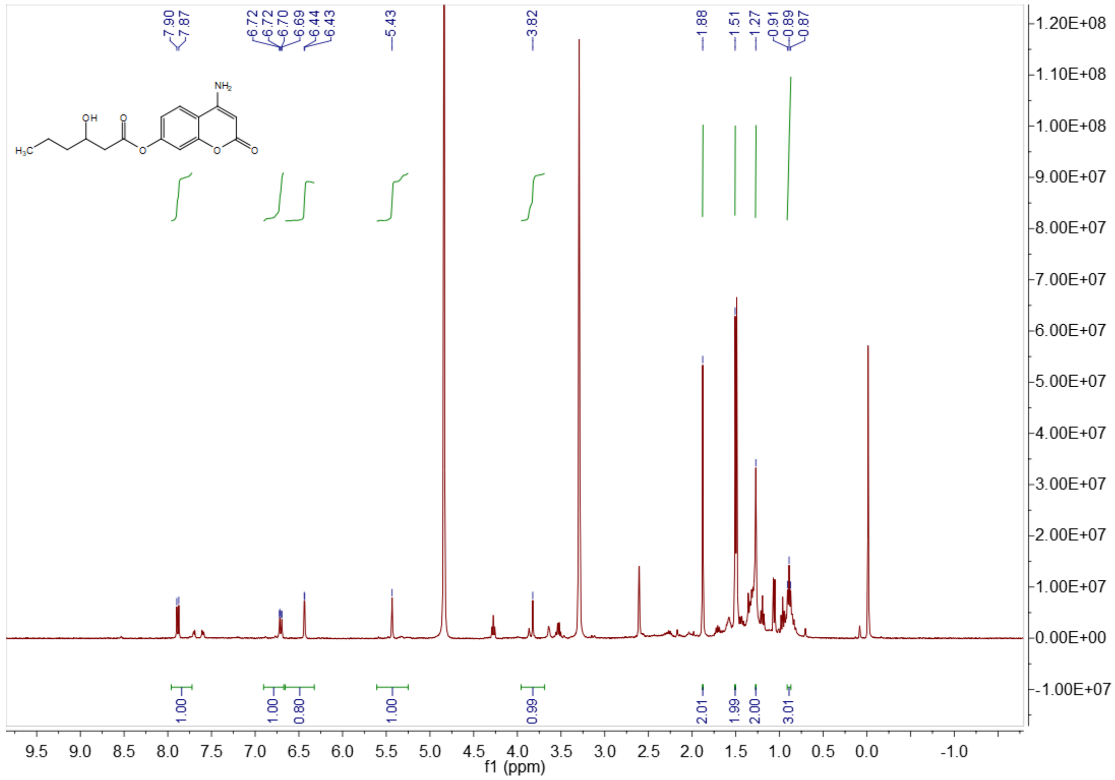

Compound 3m^{1}H NMR

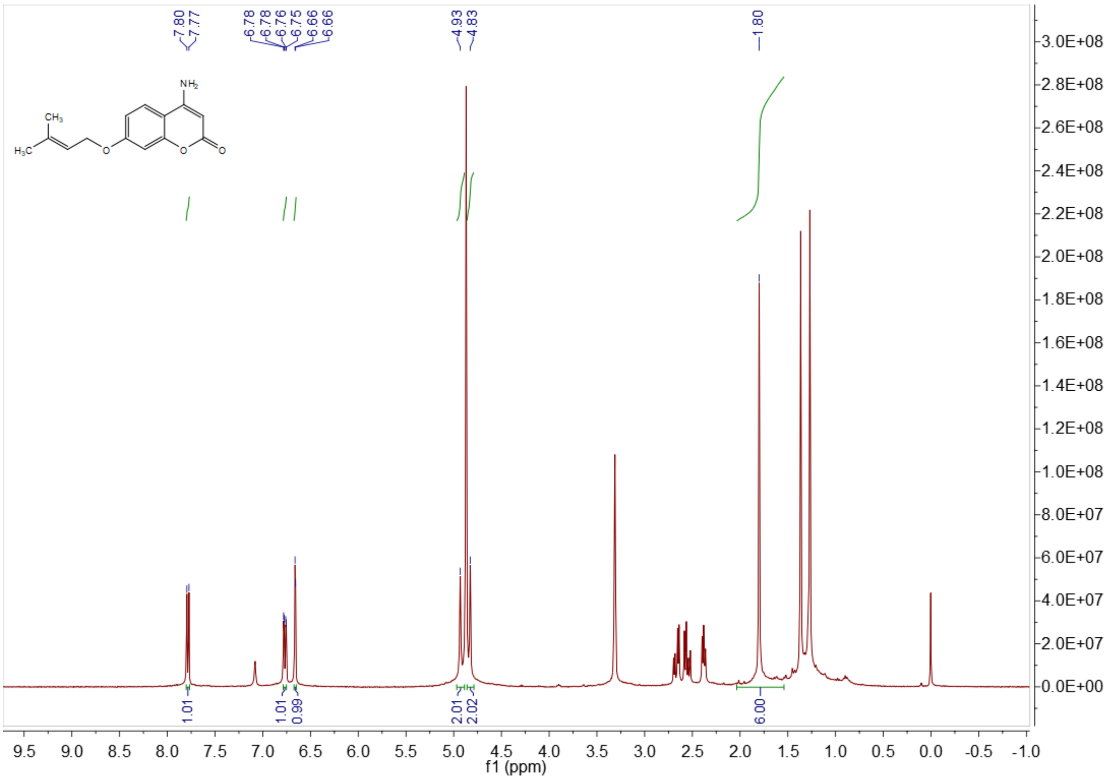

Compound 3n ^{1}H NMR

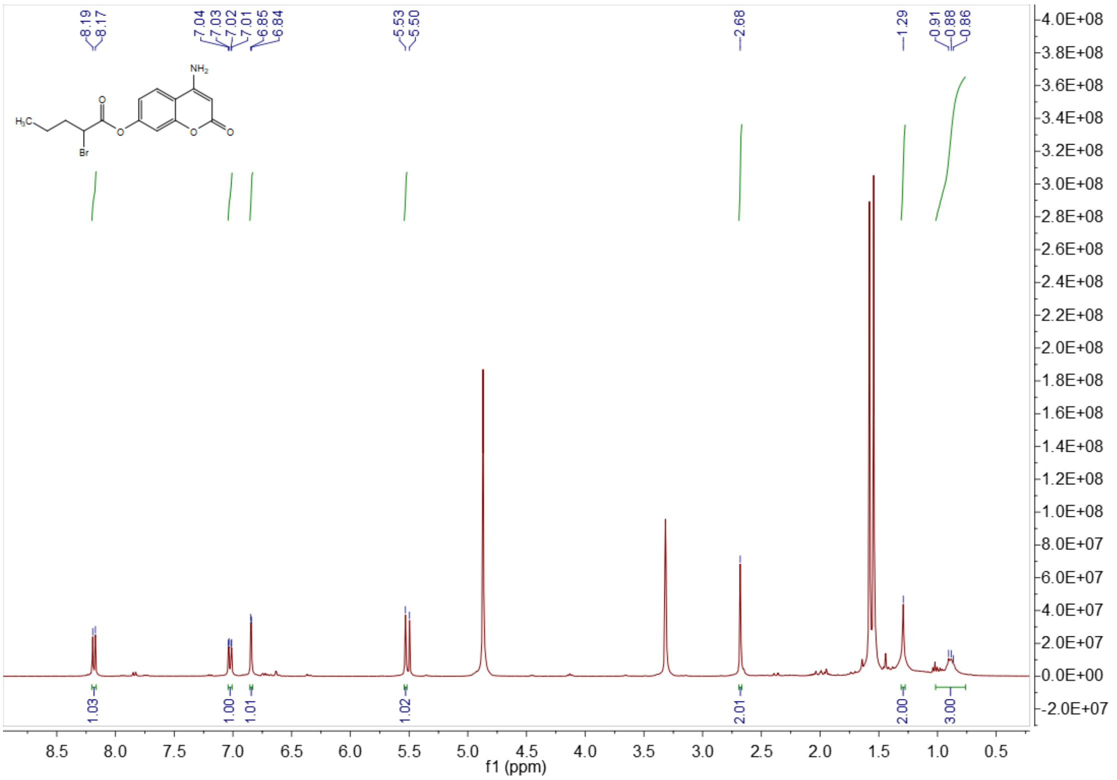

Compound 3o ^{1}H NMR

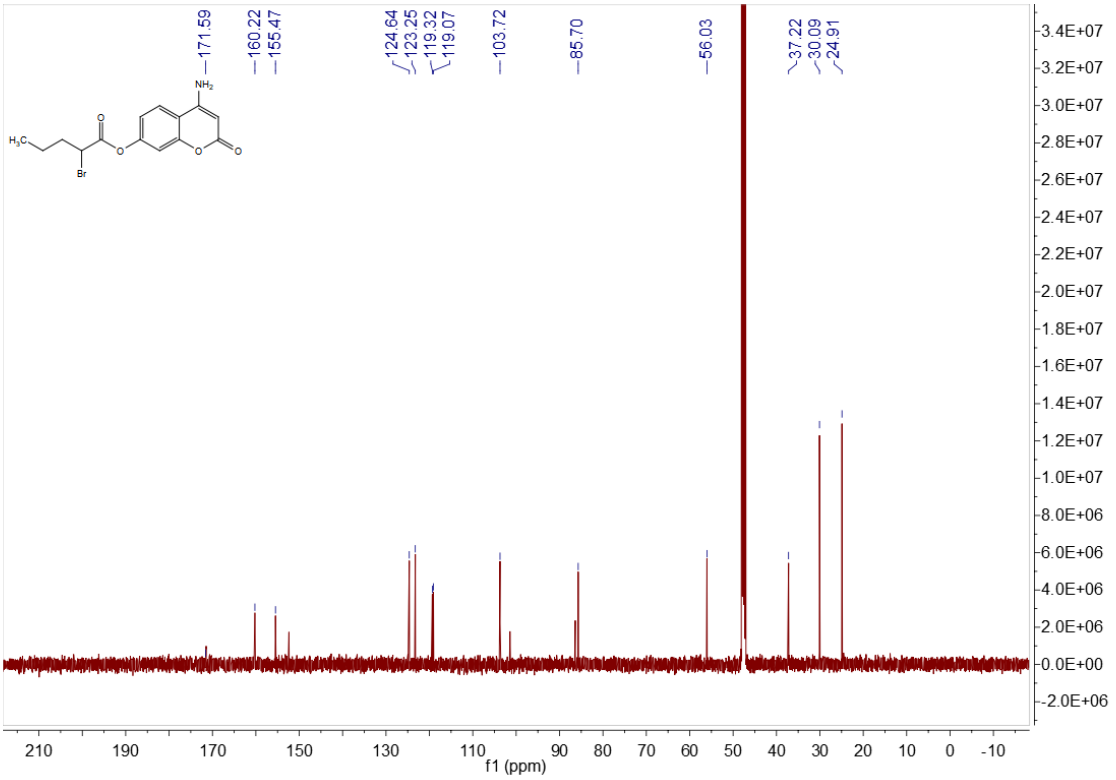

Compound 3o ^{13}C NMR

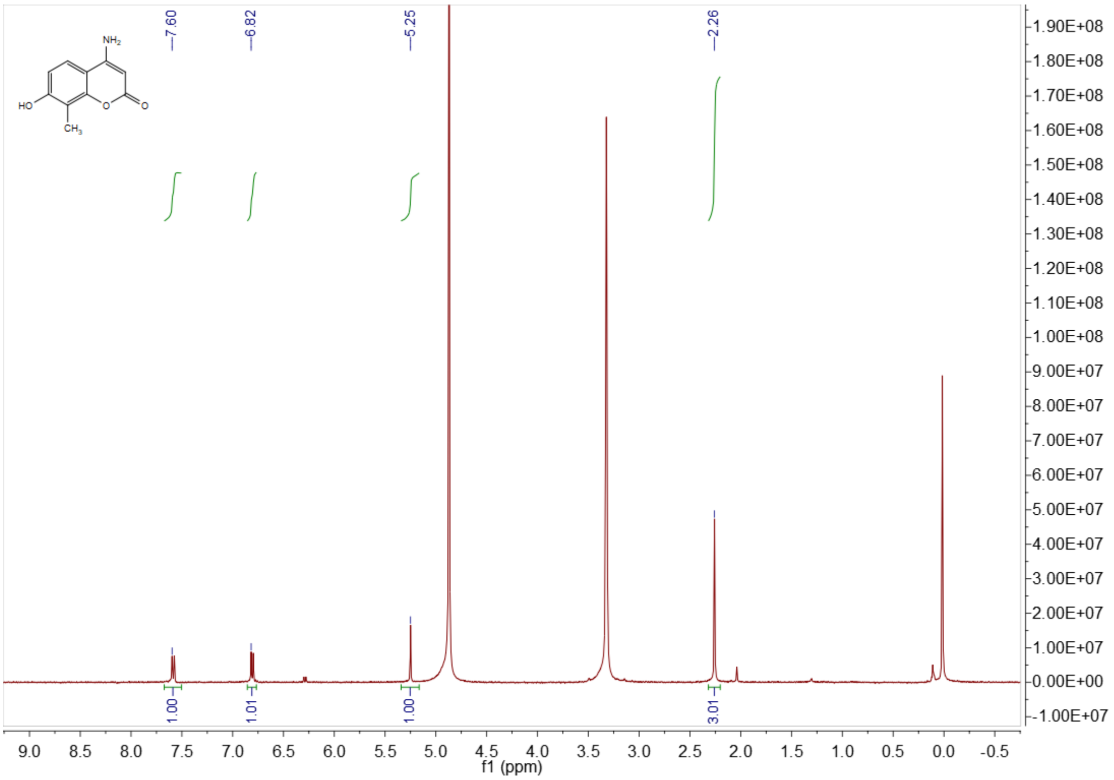

Compound 2’ ^{1}H NMR

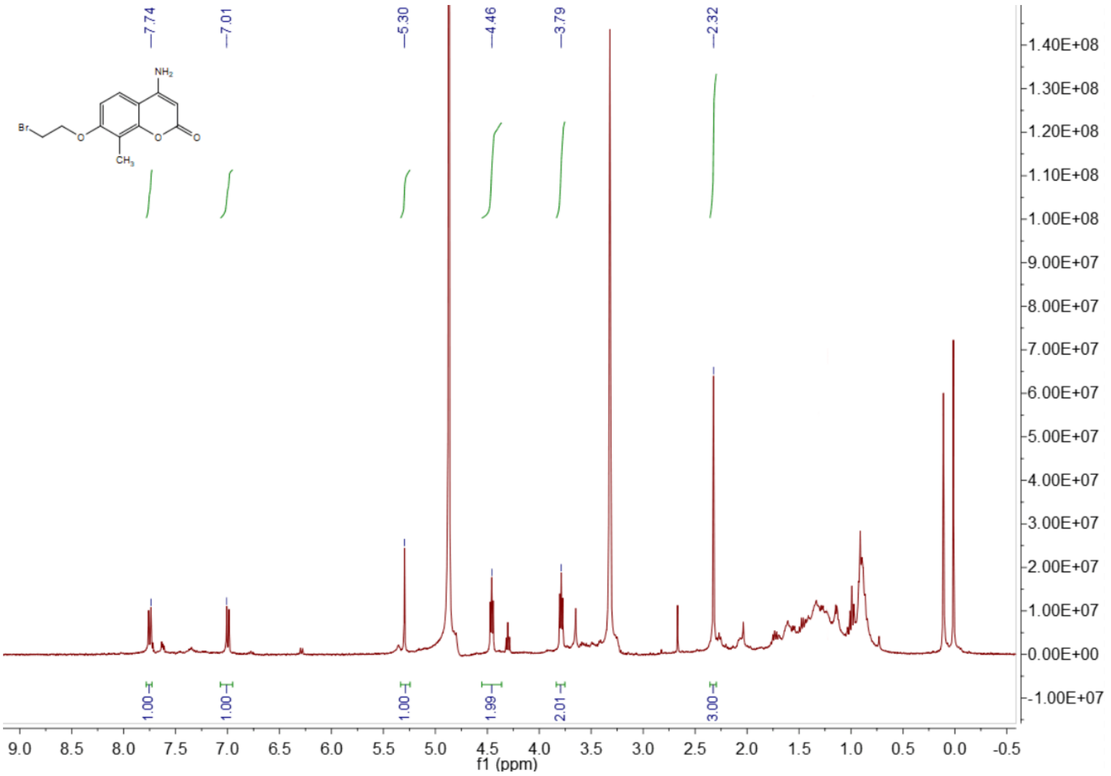

Compound 4a ^{1}H NMR

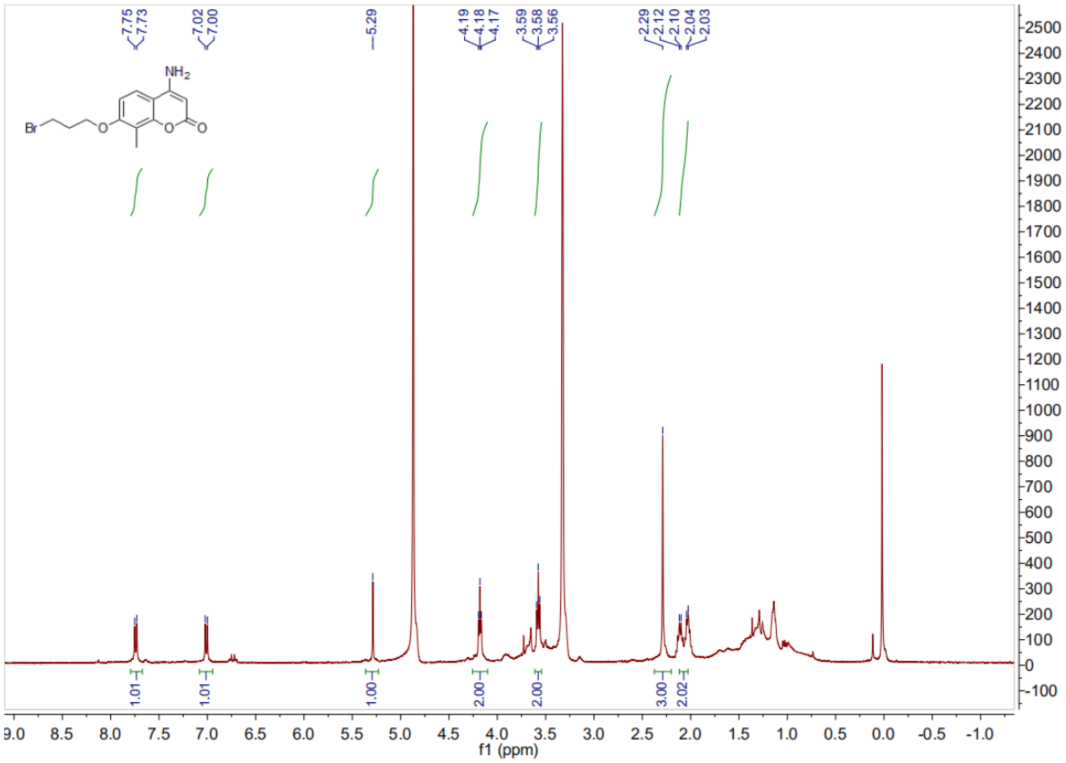

Compound 4b ^{1}H NMR

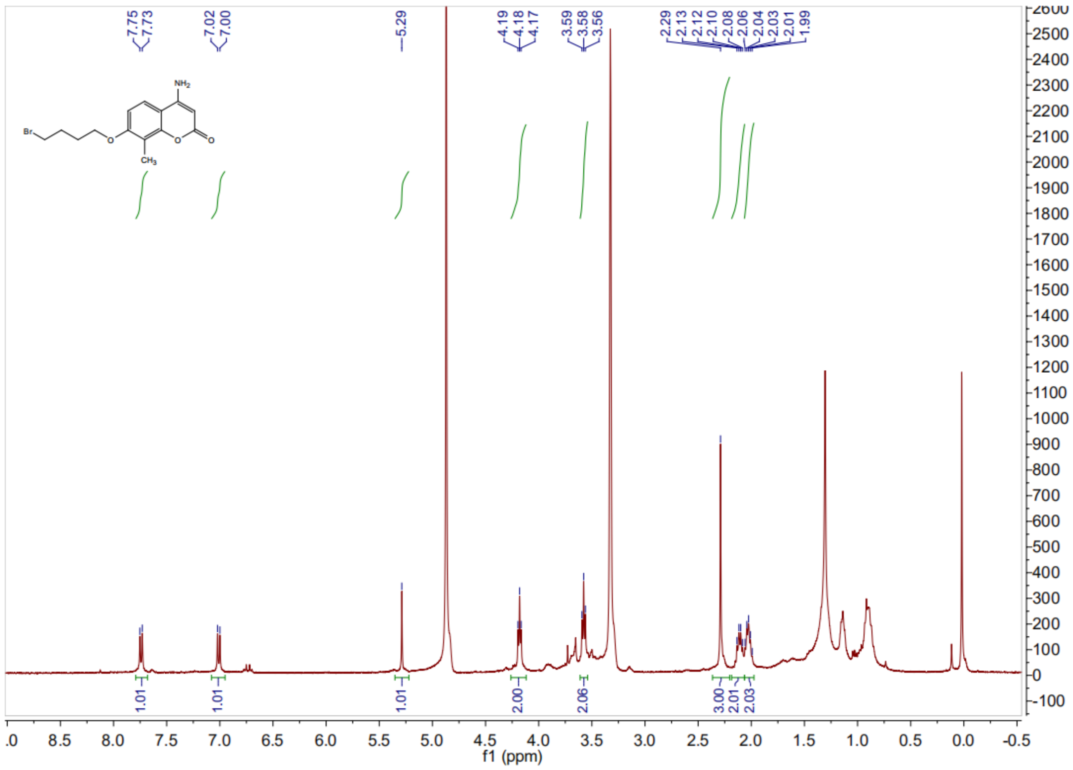

Compound 4c ^{1}H NMR

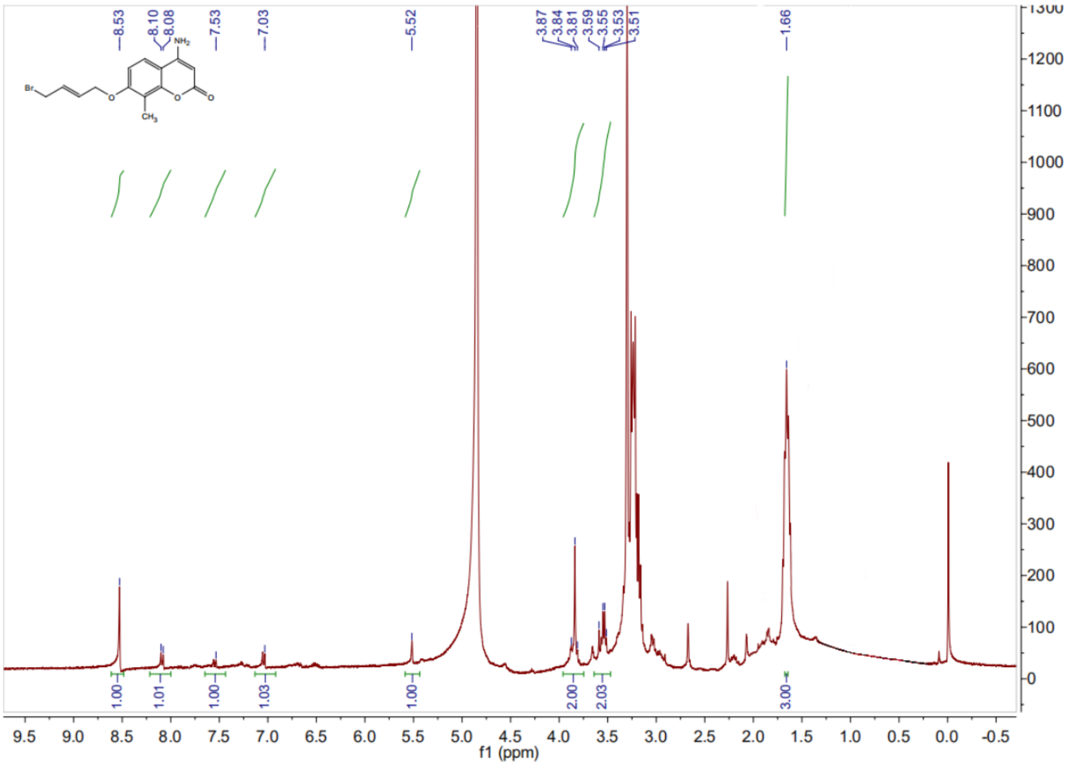

Compound 4d ^{1}H NMR

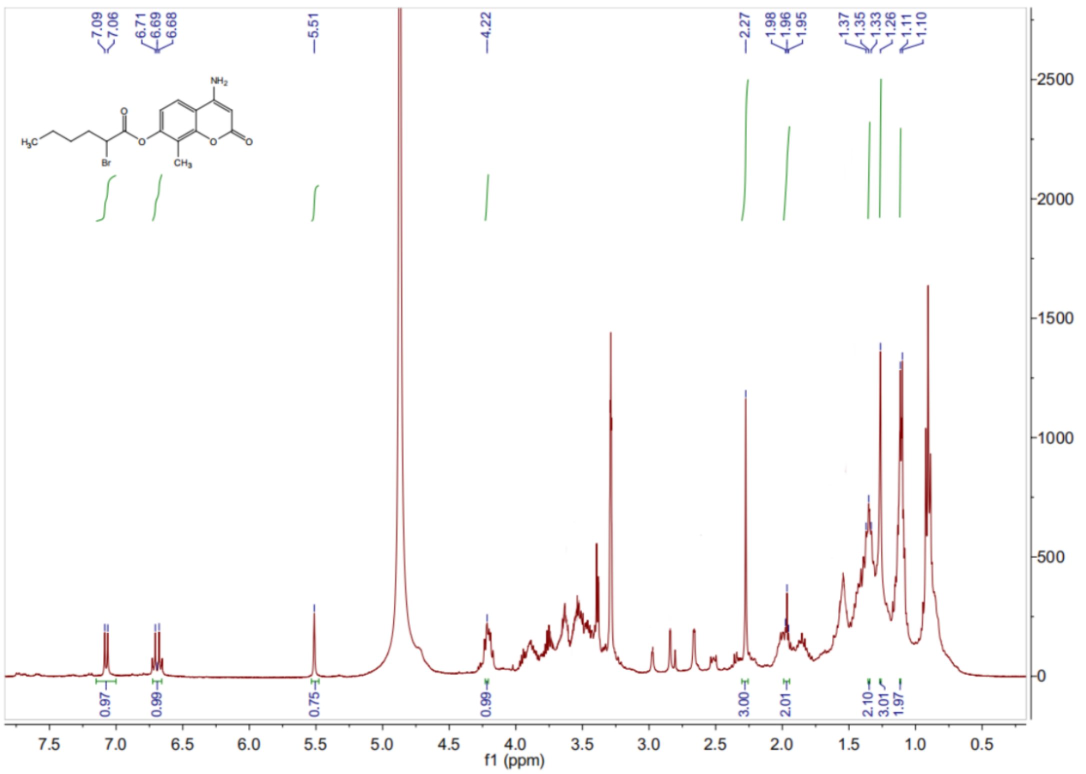

Compound 4e ^{1}H NMR

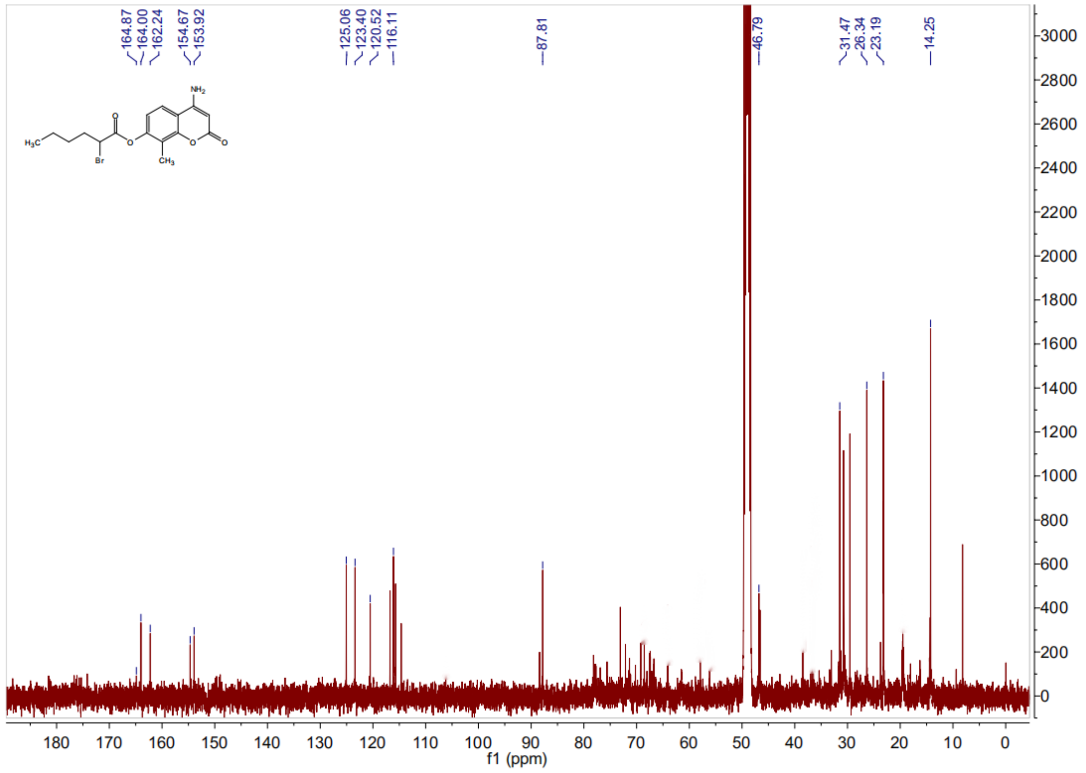

Compound 4e ^{13}C NMR

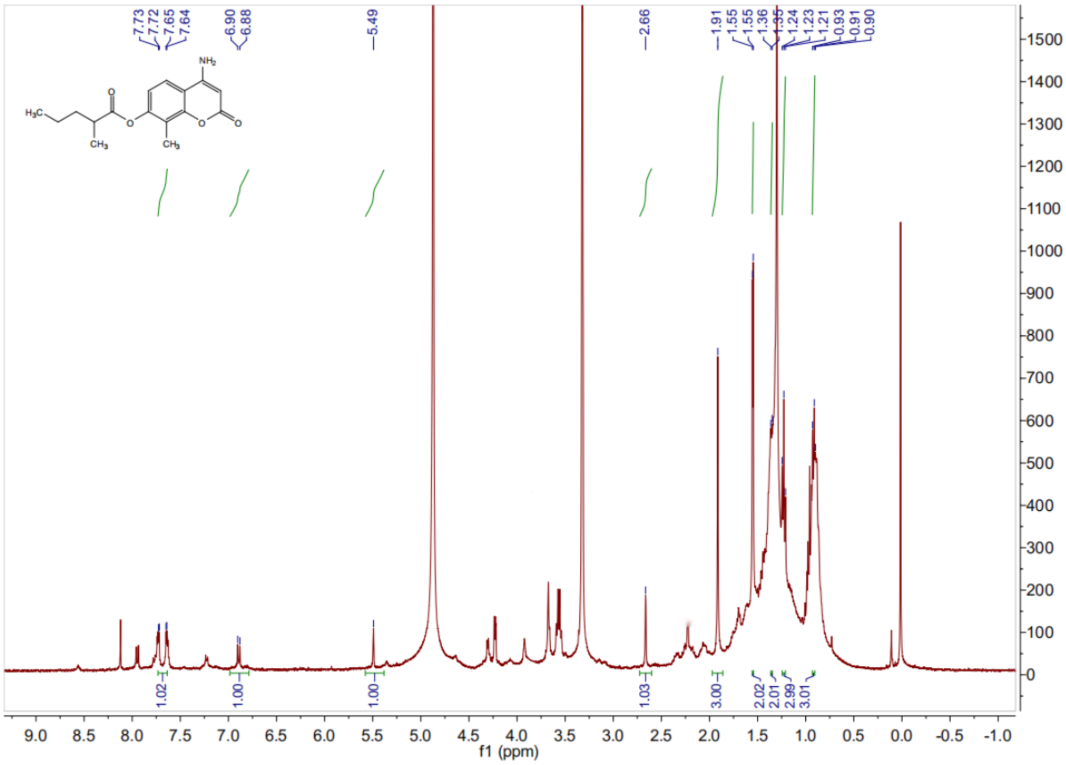

Compound 4f ^{1}H NMR

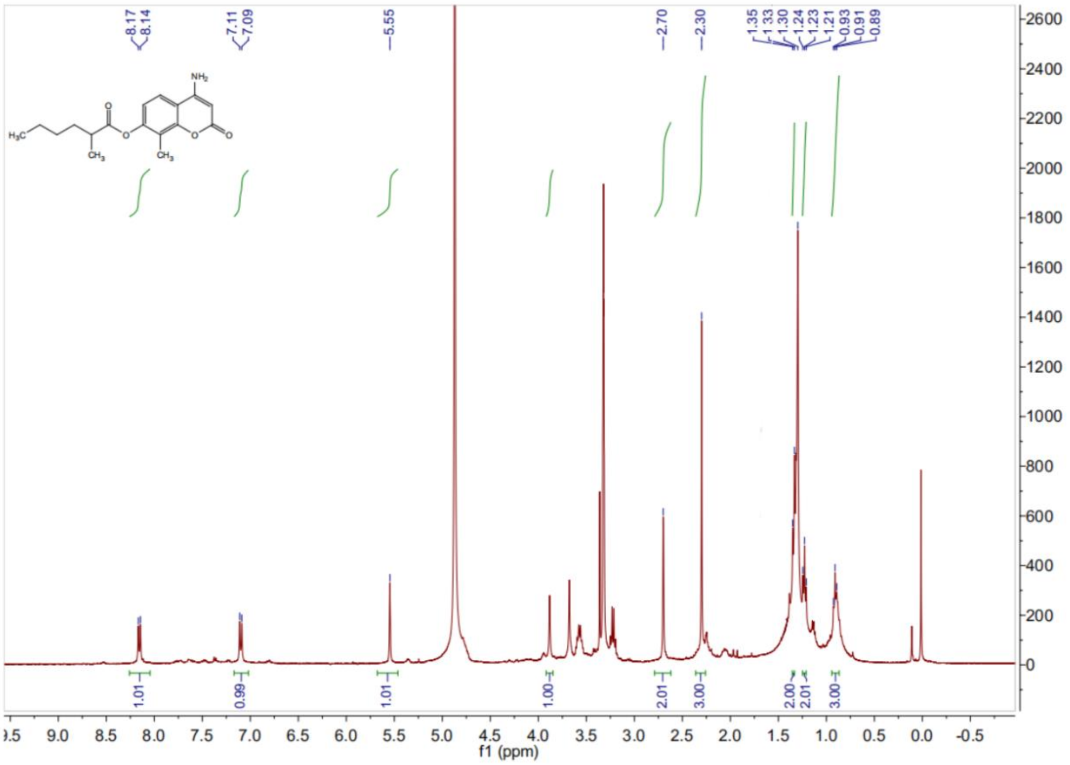

Compound 4g1H NMR

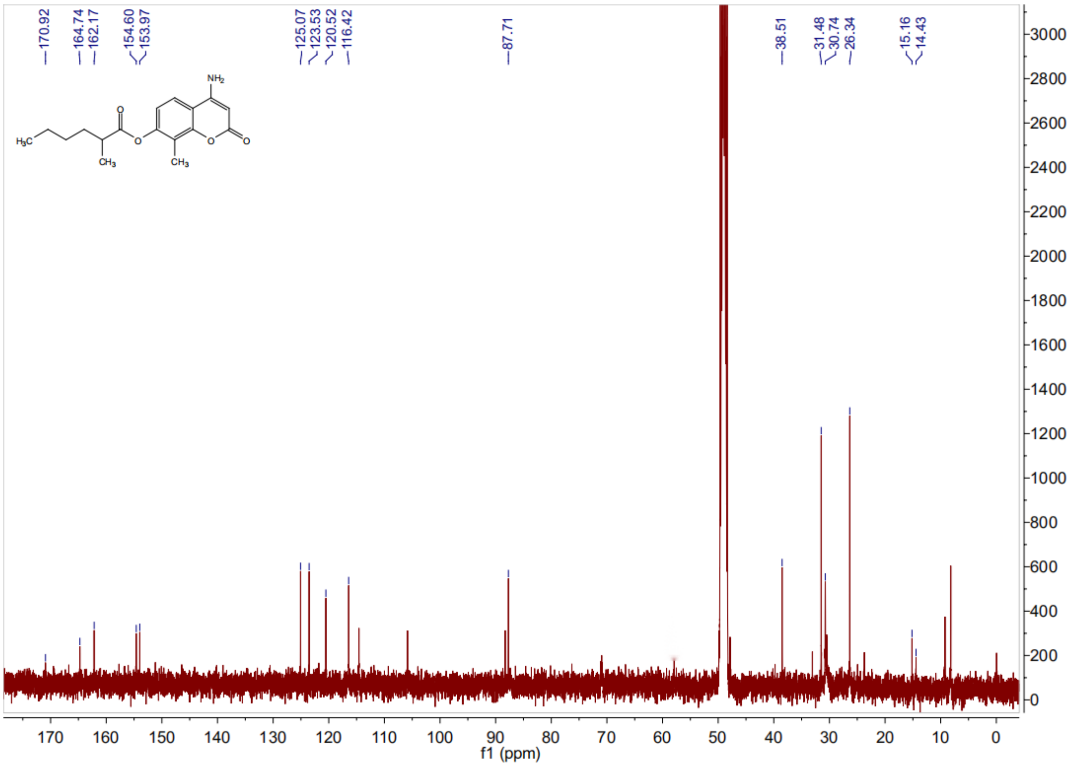

Compound 4g13C NMR

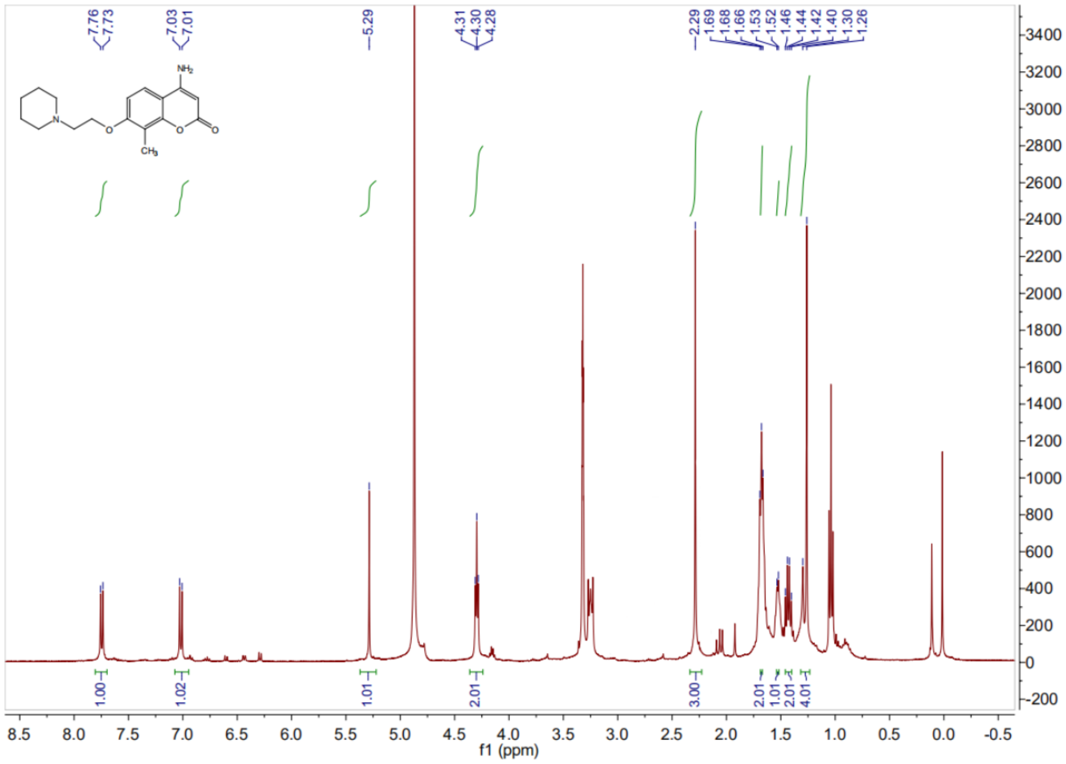

Compound 4h ^{1}H NMR

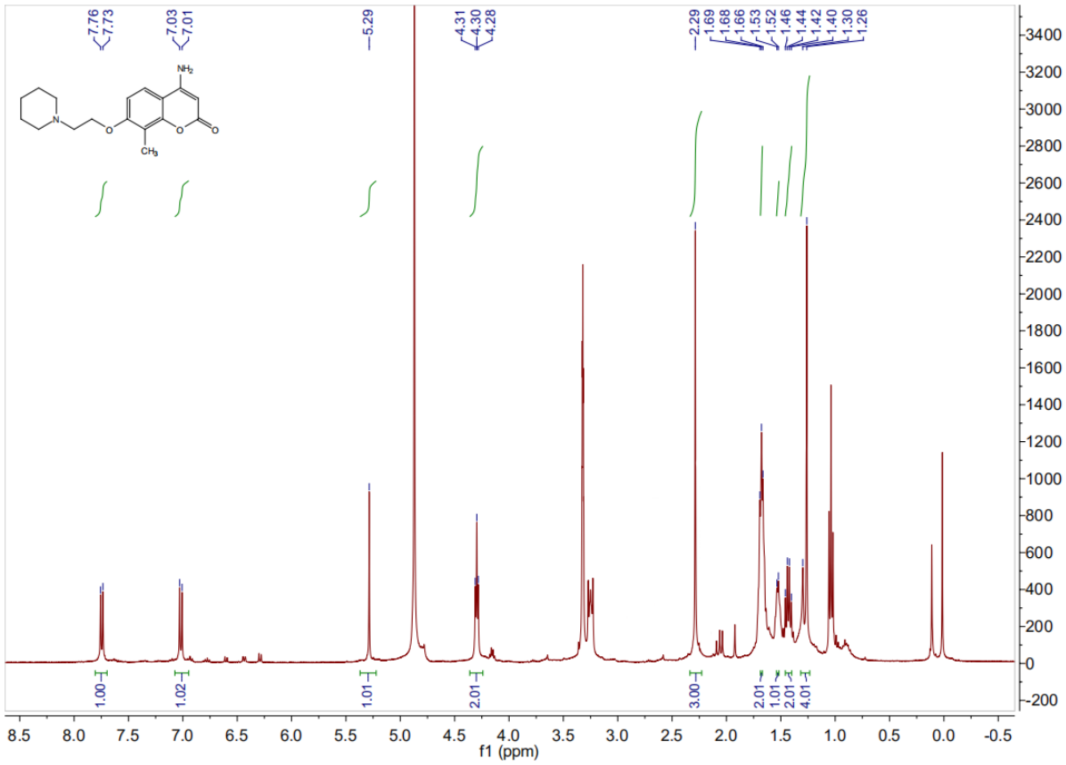

Compound 4i ^{1}H NMR

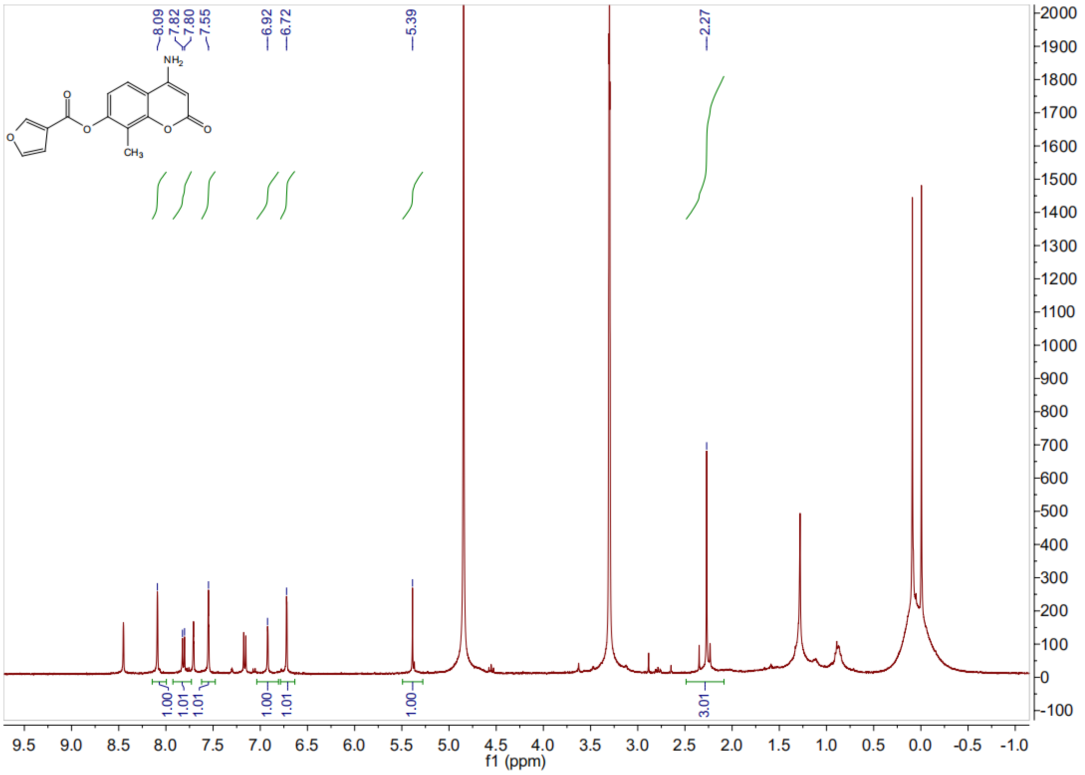

Compound 4j ^{1}H NMR

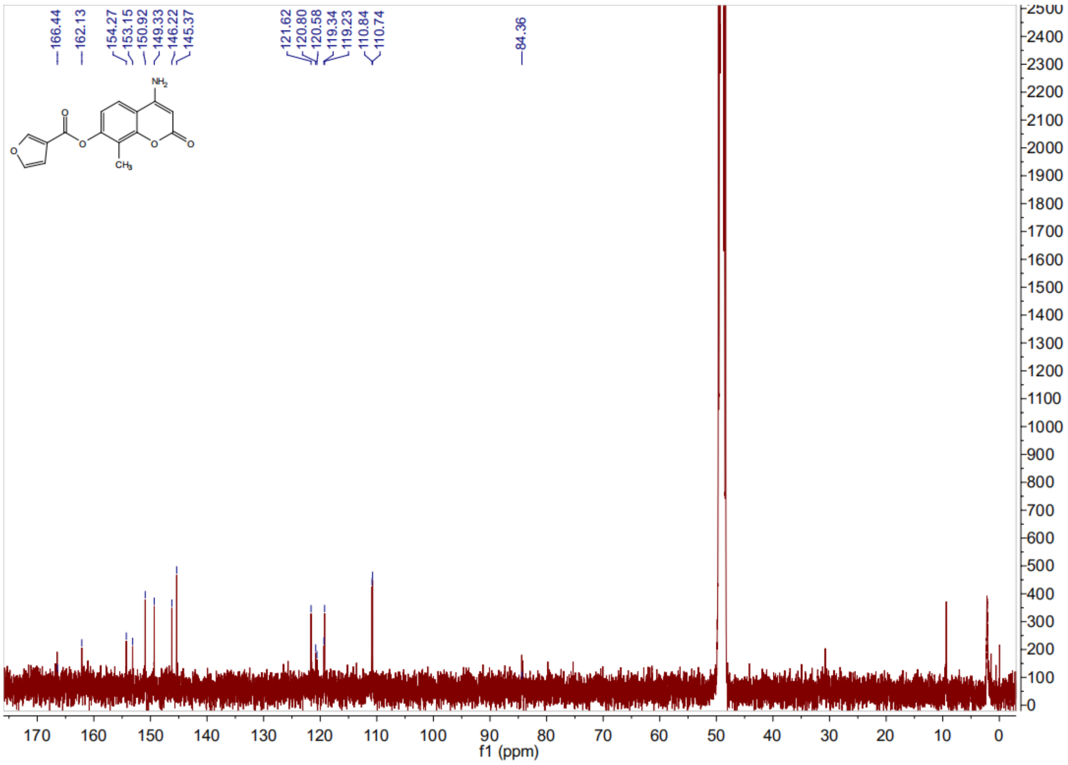

Compound 4j ^{13}C NMR

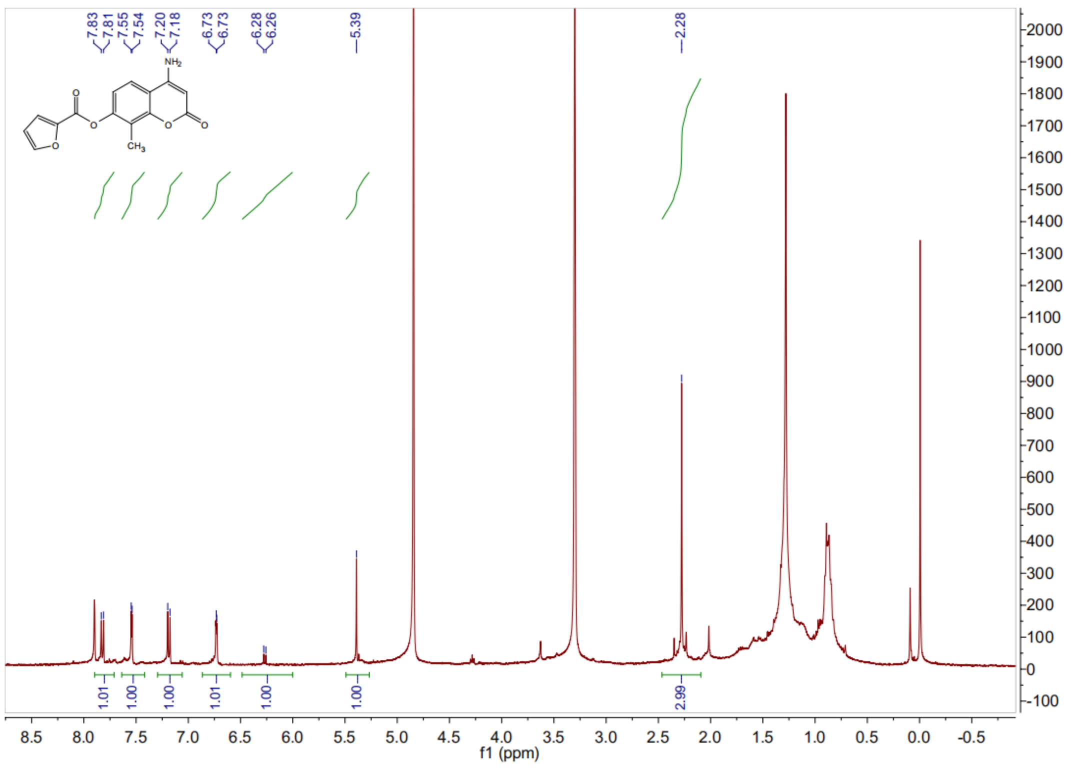

Compound 4k ^{1}H NMR

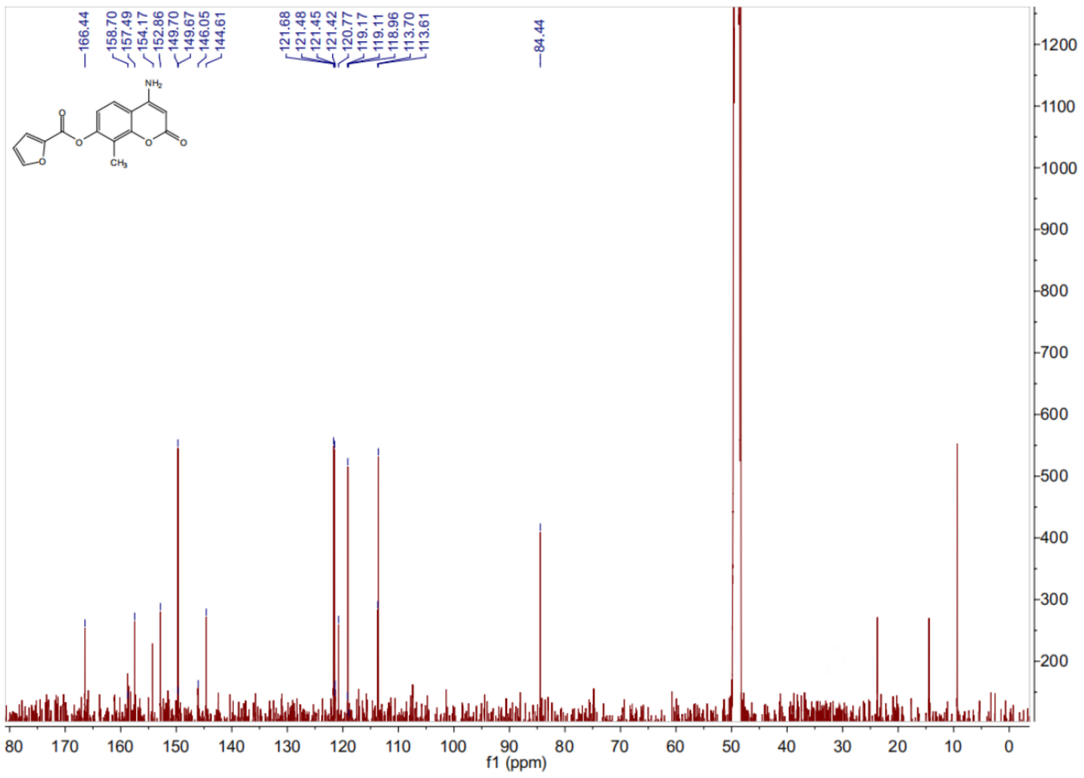

Compound 4k ^{13}C NMR

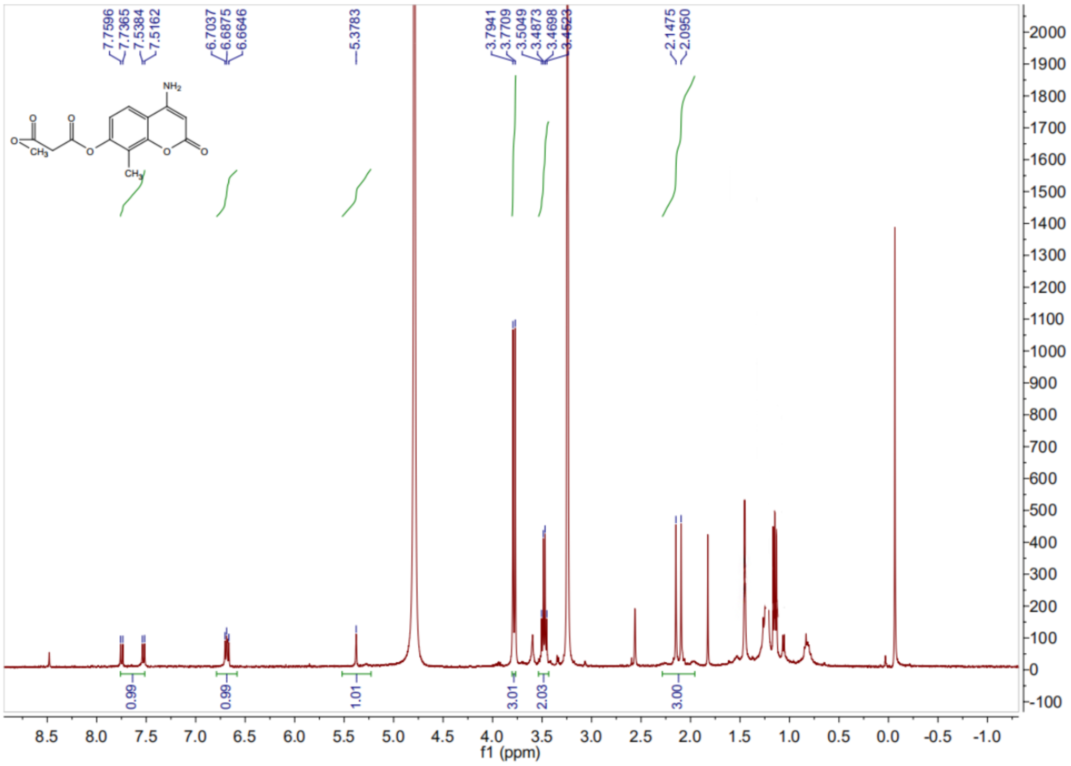

Compound 4l ^{1}H NMR

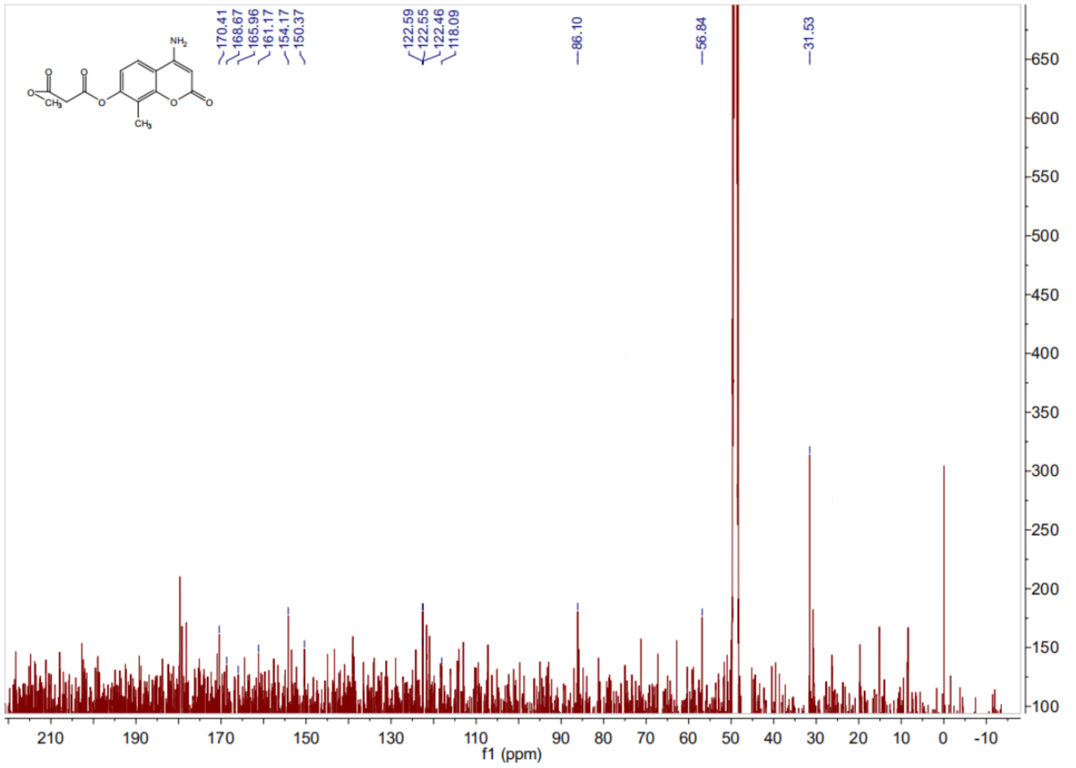

Compound 4l ^{13}C NMR

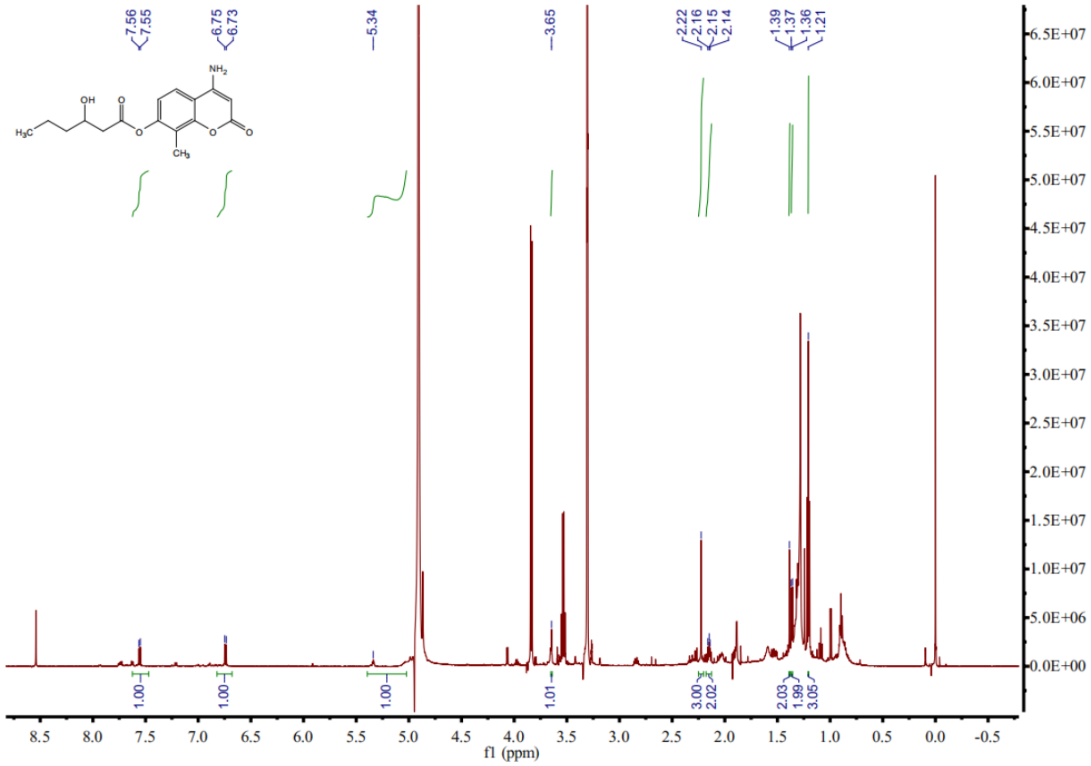

Compound 4m ^{1}H NMR

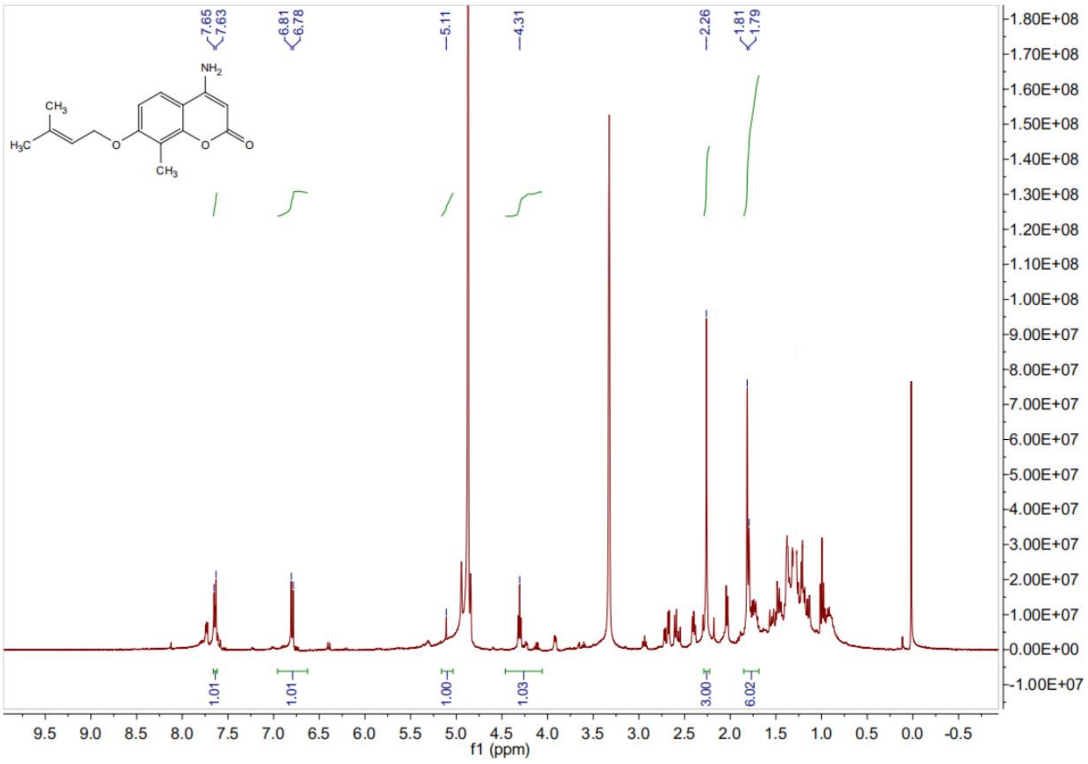

Compound 4n ^{1}H NMR

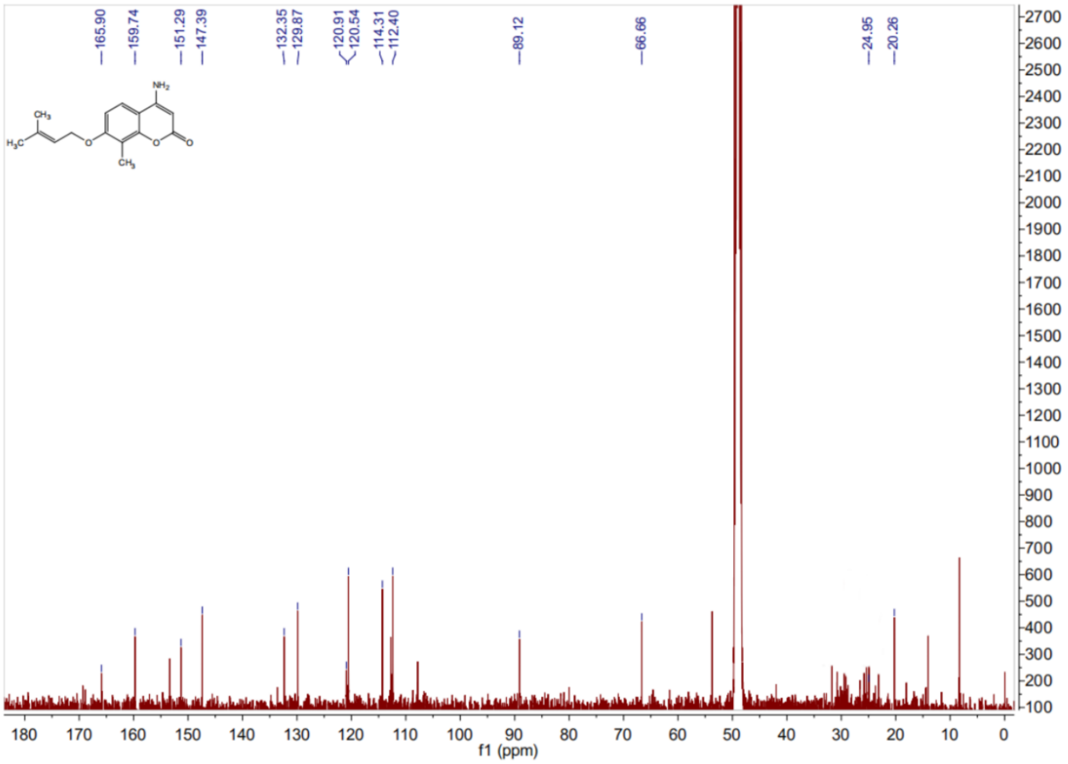

Compound 4n ^{13}C NMR

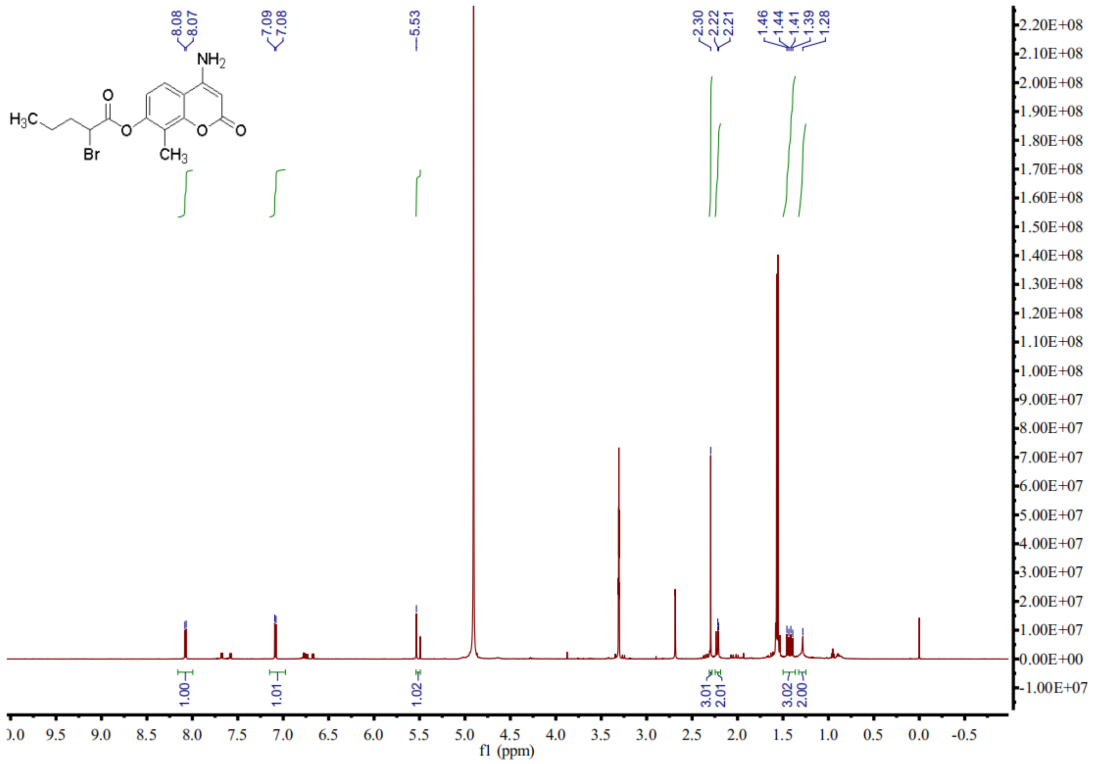

Compound 4o ^{1}H NMR

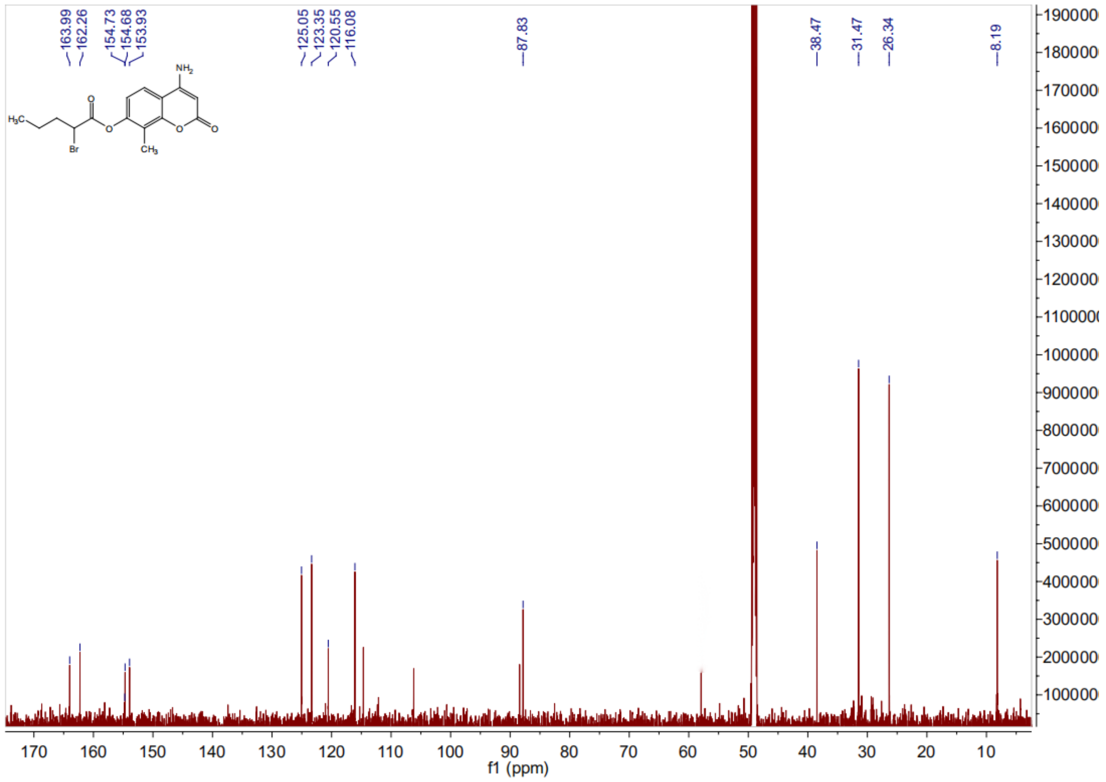

Compound 4o ^{13}C NMR
